# Longitudinal Study of the Variation in Patient Turnover and Patient-to-Nurse Ratio: Descriptive Analysis of a Swiss University Hospital

**DOI:** 10.2196/15554

**Published:** 2020-04-02

**Authors:** Sarah N Musy, Olga Endrich, Alexander B Leichtle, Peter Griffiths, Christos T Nakas, Michael Simon

**Affiliations:** 1 Institute of Nursing Science University of Basel Basel Switzerland; 2 Nursing and Midwifery Research Unit Inselspital, Bern University Hospital University of Bern Bern Switzerland; 3 Medical Directorate Inselspital, Bern University Hospital University of Bern Bern Switzerland; 4 Insel Data Science Center Inselspital, Bern University Hospital University of Bern Bern Switzerland; 5 University Institute of Clinical Chemistry Inselspital, Bern University Hospital University of Bern Bern Switzerland; 6 Health Sciences University of Southampton Southampton United Kingdom; 7 National Institute for Health Research Applied Research Collaboration (Wessex) Southampton United Kingdom; 8 LIME Karolinska Institutet Stockholm Sweden; 9 Laboratory of Biometry University of Thessaly Volos Greece

**Keywords:** patient safety, electronic health records, nurse staffing, workload, routine data

## Abstract

**Background:**

Variations in patient demand increase the challenge of balancing high-quality nursing skill mixes against budgetary constraints. Developing staffing guidelines that allow high-quality care at minimal cost requires first exploring the dynamic changes in nursing workload over the course of a day.

**Objective:**

Accordingly, this longitudinal study analyzed nursing care supply and demand in 30-minute increments over a period of 3 years. We assessed 5 care factors: patient count (care demand), nurse count (care supply), the patient-to-nurse ratio for each nurse group, extreme supply-demand mismatches, and patient turnover (ie, number of admissions, discharges, and transfers).

**Methods:**

Our retrospective analysis of data from the Inselspital University Hospital Bern, Switzerland included all inpatients and nurses working in their units from January 1, 2015 to December 31, 2017. Two data sources were used. The nurse staffing system (tacs) provided information about nurses and all the care they provided to patients, their working time, and admission, discharge, and transfer dates and times. The medical discharge data included patient demographics, further admission and discharge details, and diagnoses. Based on several identifiers, these two data sources were linked.

**Results:**

Our final dataset included more than 58 million data points for 128,484 patients and 4633 nurses across 70 units. Compared with patient turnover, fluctuations in the number of nurses were less pronounced. The differences mainly coincided with shifts (night, morning, evening). While the percentage of shifts with extreme staffing fluctuations ranged from fewer than 3% (mornings) to 30% (evenings and nights), the percentage within “normal” ranges ranged from fewer than 50% to more than 80%. Patient turnover occurred throughout the measurement period but was lowest at night.

**Conclusions:**

Based on measurements of patient-to-nurse ratio and patient turnover at 30-minute intervals, our findings indicate that the patient count, which varies considerably throughout the day, is the key driver of changes in the patient-to-nurse ratio. This demand-side variability challenges the supply-side mandate to provide safe and reliable care. Detecting and describing patterns in variability such as these are key to appropriate staffing planning. This descriptive analysis was a first step towards identifying time-related variables to be considered for a predictive nurse staffing model.

## Introduction

Determining appropriate nursing staff numbers and skill mixes in hospital units is vital to both ensure quality of care [1-4] and maintain health care budgets [5]. Understaffing or poor skill mixes can lead to adverse patient outcomes, while overstaffing can lead to budgetary overruns and ultimately close hospitals. In economic terms, the relationship between patients and nurses is one of supply and demand, respectively, representing the amount of care required by the patients versus the nursing staff’s capacity to provide that care.

Undoubtedly, care demands and staffing requirements vary widely across departments, units, seasons, months, days of the week, shifts (morning, evening, or night), and even hours [6-8]. Each unit’s patient count fluctuates with patient turnover resulting from admissions, discharges, and transfers within and between units [9-12]. In turn, turnover affects the volume of nurses’ clinical and administrative duties [13,14].

Another notable factor is patient acuity, or the amount of time each patient requires. Newly admitted or transferred patients tend to have high levels of acuity because they require baseline assessments and treatments [15,16]. Specific patient characteristics, including demographics (eg, age, gender, family support, socioeconomic factors), personal background, diagnoses, and treatment regimes, can also increase acuity. For example, patients who are older [17-19], lack family support [18], or have limited knowledge of their health condition(s) [20] have more complex care needs, with strong implications for nurse staffing. Depending on each shift’s patient numbers and combined acuity, nursing workloads can vary across and within wards. Meeting their specific needs requires an appropriate number and skill mix of nurses: registered nurses (RNs), licensed practical nurses (LPNs), and unlicensed personnel. Some studies have used nursing staff with or without qualification in their analyses [21,22]. In Switzerland, RNs typically represent the major proportion of hospital nursing staff; however, direct and indirect patient care typically involves collaborations between nurses with a broad range of qualifications.

To date, the majority of research about nurse staffing has fit into two categories: longitudinal studies conducted over relatively long periods (eg, years) or across locations and cross-sectional studies often conducted across multiple locations. However, in both cases, large-scale views fail to capture shift-level or daily variations in either supply or demand. This lack of detail limits the understanding of the association between staffing, patient turnover, and relevant human and economic outcomes [7]. Noting that these limitations severely limit the value of research findings for staffing guidelines [22], studies have begun to highlight the advantages of both focusing on unit-level dynamics and using hospital record data longitudinally rather than cross-sectionally [21,23,24].

Every nursing unit manager’s job includes assuring patient safety and quality of care every hour of the day. However, due to the noted limitations and considering the principle that nurse-patient relationships occur on the individual level [25], previous studies have offered a limited view of the small-scale supply and demand dynamic of nursing workload. Nurse staffing planners are particularly challenged by demand-side variability, which occurs over very short periods. Therefore, to both optimize staffing levels (ie, maintain levels that will allow safe patient care while minimizing personnel costs) and develop reliable staffing guidelines, it is necessary to record and explore fluctuations in nursing workload throughout the day rather than simply considering daily or shift averages.

Therefore, this study’s overall aims were to describe the supply and demand dynamics of nursing care and identify mismatches between supply and demand from a longitudinal perspective. Specifically, in 30-minute increments, across a range of units in a Swiss University Hospital (Inselspital, Bern), we describe every recorded change in patient numbers (ie, care demand); nurse numbers (ie, supply); patient-to-nurse ratios for the various nurse groups; extreme supply and demand mismatches; and patient turnover (ie, numbers of admissions, discharges, and transfers).

## Methods

### Study Design and Setting

This retrospective, descriptive, observational study used routinely collected patient data from the Inselspital University Hospital, Bern, Switzerland. As one of Switzerland’s five University Hospitals, the Inselspital treats approximately 48,000 inpatients annually [26]. Only inpatient units with data for the full 3 years were included. Our data were drawn from 10 departments: (1) Internal Medicine; (2) Cardiology & Cardiovascular Surgery; (3) Orthopedics & Plastic Surgery; (4) Neurology, Neurosurgery, Otolaryngology, Head and Neck Surgery, & Ophthalmology; (5) Visceral Surgery and Medicine, Gastroenterology, Thoracic Surgery, & Pulmonology; (6) Dermatology, Urology, Rheumatology, & Nephrology; (7) Hematology & Oncology; (8) Maternity & Gynecology; (9) Pediatrics; and (10) Intensive Care. Full data (2015–2017) were available for all these departments for the full study period.

### Participants

#### Patients

All inpatients were included. No further specific eligibility criteria were applied.

#### Nurses

All staff providing direct or indirect nursing care were considered in the analysis, independent of educational background. We divided nursing staff into five groups: RNs, including nurses in supervisory positions (group 1); LPNs (group 2); others, including unlicensed and administrative personnel (group 3); students (group 4); and external nurses (agency staff; group 5). In Switzerland, RNs complete a 3-4–year tertiary professional or university-based education (group 1). Unlike in other countries, Switzerland also offers 3 years of secondary-level professional training for nursing assistants (group 2). Group 3 included unlicensed personnel, including nursing aides with minimal education or training, and administrative staff. Group 4 included both nursing and medical students working as nursing aides.

### Data Sources and Variables

We extracted our data from two sources: the tacs nurse staffing system (ie, datasheets organized in a relational database) and medical discharges. From tacs, we extracted four care-relevant factors: nurses, patients, activities, and care-related working hours. The tacs system records time allocations provided by every nurse at the end of every shift. In addition to administrative work, teaching duties, and continuous education, each record specifies the time devoted to each patient’s direct and indirect care. No further details about the type of activity such as medication, mobility, or respiratory therapy are currently available. Nurses’ absences such as holidays, illnesses, or accidents are also recorded. Patient turnover information is provided with the nursing unit, date, and time, as well as whether inpatient or outpatient services were provided. Finally, medical discharge data include patient demographics, admission and discharge details, and diagnoses. Each data record identifies the relevant unit and includes identifiers for the nurse (and her or his contract) and each case (patient) cared for during that shift. Based on these identifiers, the 5 datasheets were linked at the patient, nurse, and unit levels in a single dataset. Then, all patient, nurse, and unit identifiers were deidentified, leaving only department names. Table 1 describes the 17 variables used in the analysis.

**Table 1:** Description of the 17 variables used for the current analysis, listed in alphabetical order.

Variable and short description			Source^a^
**Type of activity performed by the nurse**			
	Indirect and direct care		A^b^
	Administrative work		A
	Teaching assignments		A
	Continuous education		A
	Absences (ie, holidays, illnesses, accidents)		A
**Admission date**			
	Patient’s hospital admission date		P^c^
**Admission time**			
	Patient’s hospital admission time		P
**Age**			
	Patient’s age at admission		M^d^
**Case identifier**			
	Unique code for the patient’s case (deidentified to “Patient1”, “Patient2”, etc)		A, P, M
**Departments of the Inselspital, Bern University Hospital**			
	Cardiology & Cardiovascular Surgery		A, M
	Neurology, Neurosurgery, Otolaryngology, Head and Neck Surgery, & Ophthalmology		A, M
	Intensive Care		A, M
	Pediatrics		A, M
	Dermatology, Urology, Rheumatology, & Nephrology		A, M
	Visceral Surgery and Medicine, Gastroenterology, Thoracic Surgery & Pulmonology		A, M
	Internal Medicine		A, M
	Maternity & Gynecology		A, M
	Orthopedics & Plastic Surgery		A, M
	Hematology & Oncology		A, M
**Contract identifier**			
	Unique code for each nursing position/contract (a nurse can have multiple contracts within the hospital involving various qualifications or working units), which was deleted after merging		A, N^e^, W^f^, M
**Date**			
	Working date of the nurse		A, W
**Discharge date**			
	Patient’s hospital discharge date		P, M
**Discharge time**			
	Patient’s hospital discharge time		P
**End time**			
	Time at which the nurse stopped work for the shift or started a break		W
**Group (classifications of nurse qualifications)**			
	Registered nurses		N
	Licensed practical nurses		N
	Others (eg, unlicensed and administrative personnel)		N
	Students		N
	External nurses		N
**Main diagnosis**			
	ICD-10-GM^g^ codes for the patient’s main diagnosis		M
**Nurse identifier**			
	Unique code for a nurse (deidentified to “Nurse1”, “Nurse2”, etc)		A, N, W
**Start time**			
	Time at which the nurse began work or returned from a break		W
**Transfer date**			
	Transfer date of the patient within and between departments		P
**Transfer time**			
	Transfer time of the patient within and between departments		P
**Unit identifier**			
	Unique code for the unit (deidentified to “Unit1”, “Unit2”, etc within each department)		A, N, P

^a^Source: nurse staffing system (tacs) or medical discharge data.

^b^A: nurse staffing system activity data.

^c^P: nurse staffing system patient data.

^d^M: medical discharge data.

^e^N: nurse staffing system nurse data.

^f^W: nurse staffing system working hours data.

^g^ICD-10-GM: 10th revision of the International Statistical Classification of Diseases and Related Health Problems, German Modification.

#### Ethical Considerations

Our acquisition of data from the Inselspital (University Hospital of Bern) was outside the purview of the Cantonal Ethic Commission of Bern based on the Swiss legislation on research with humans (Req-2016-00618). All data involving patients, nurses, and units were deidentified.

### Statistical Analyses

All statistical analyses were conducted using R, version 3.5.1 for Mac OS and Linux [27]. To handle and manipulate the data, we used the purrr [28], dplyr [29], tidyr [30], and data.table [31] packages. To manipulate time and date, we used the lubridate [32], chron [33], and padr [34] packages. To create plots, we used the ggplot2 [35] and scales [36] packages.

#### Linking Procedure

Data were merged based on 6 key variables: patient identifier, nurse identifier, contract identifier, unit identifier, time, and date. First, a subset of activity data was created for data on inpatient units and direct and indirect care. This subset was then divided into nurse and patient activity fields, and any duplicate records were deleted. Each nurse’s activities were merged first with her or his other data, then with the data she or he supplied regarding time use, contract identifier, and date. Contract identifiers were deleted, and nurses and units were deidentified. Similarly, each patient’s activity data were merged first with their other data, then with medical controlling data by case identifier. To maintain consistent patient counts, healthy newborn babies in the Maternity & Gynecology department were excluded. Patients and units were then deidentified. Finally, the merged nurse and patient data were expanded to allow assessment of the number of nurses, patients, admissions, discharges, and transfers in 30-minute increments.

#### Descriptive Overview

For each department, the total numbers of units and patients were recorded. Mean (SD) and median (interquartile range [IQR]) were calculated for patient age. Length of stay (LOS) in days was computed by subtracting the discharge date from the admission date. Median (IQR) was calculated for LOS and for the number of patients per day per unit. Finally, for each department, we identified the two most common diagnoses by incidence (%).

#### Number of Patients

To keep computational complexity to a reasonable limit, patient numbers (ie, demand) were calculated at 30-minute intervals. Alternative increments (20, 40, and 60 minutes) had no relevant effect on the results. However, as patients rarely arrive or leave at shift divisions and some do not stay on the unit for one full shift, a short interval length ensures precise patient numbers. Unit-level calculations correspond to every 30-minute interval (ie, 48 data points per day) totaling 153,792 points per unit during the study period.

#### Number of Nurses

As with the number of patients, the number of nurses in each group (ie, supply) was calculated for each 30-minute increment. As nurses may work only half shifts or overlapping shifts, this increment length ensured precise numbers. For each of the 48 daily data points for the 3 years covered by the study, the numbers of RNs, LPNs, and other staff were calculated. Unfortunately, as external nurses and students are not classified as typical employees, our datasets included no breakdown of their time allocation. As only daily information was available for these groups, their total numbers over the 3-year study period as well as their daily means and medians were calculated to provide an image of their effects across each unit.

#### Patient-to-Nurse Ratio

The patient-to-nurse ratio was computed by dividing the number of patients by the number of nurses at every data point. Along with numbers of patients and nurses, patient-to-nurse ratios were plotted separately for each day’s 48 data points and for each day of the week using each unit’s and each department’s mean with CIs.

#### Extreme Mismatch Between Supply and Demand

Additionally, for three key time points of each day, namely at 2 am, 10 am, and 6 pm, the unit-level median (IQR) patient-to-nurse ratio was calculated for weekdays and weekends and divided by department. Further, we calculated when 50% more or less work was required per nurse for every data point and unit based on the median patient-to-nurse ratio. Two variables were created:


extreme lower threshold = median - (median/2)

extreme higher threshold = median + (median/2)

These arbitrary cut-offs were set to illustrate extreme staffing situations. Extreme staffing situations are important to identify times where supply and demand do not match (eg, the demand is too high for the given supply or vice versa). This is an indicator of whether supply and demand are staying within the “normal” range throughout the year. For the 3-year study period, the percentages of data points falling far below or far above the thresholds were calculated. Medians (IQRs) and extreme higher and lower thresholds (% of data points) were plotted with bar charts to highlight variations in patient-to-nurse ratios. Graphics and calculations were computed separately for nurse groups 1 (RNs), 2 (LPNs), and 3 (others).

#### Patient Turnover

For every unit, the numbers of admissions, discharges, and transfers were computed for every 30-minute data point during the 3-year study period. Admission corresponds to any entry of a patient to the hospital and discharge to any exit from the hospital. Transfers, corresponding to movement of admitted patients from one unit to another, were divided into “Transfers in” and “Transfers out” of each relevant unit. As the units were of different sizes, the numbers of admissions, discharges, and transfers in and out were divided by the number of patients present at each specific data point. This allowed us to obtain a ratio for patient turnover that could be plotted on the same scale for all the units. Finally, vertical bar charts were created for weekdays and weekends by calculating the mean of the units for the departments. The left side of the vertical bar charts represents patients leaving the unit (ie, discharges and transfers out), while the bars on the right side represent incoming patients (ie, admissions and transfers in).

## Results

### Linking Procedure

**Figure 1:**
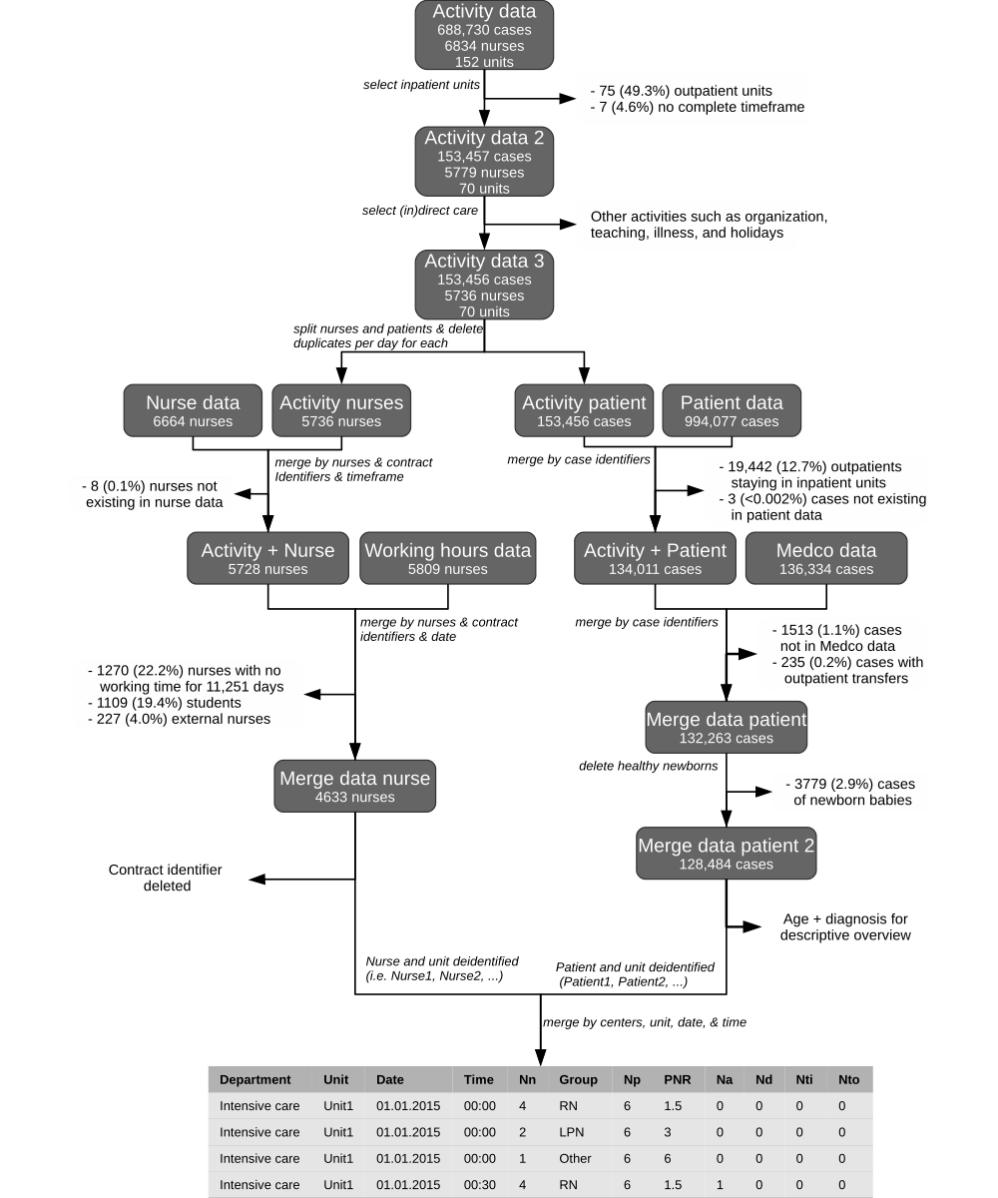
Process to link the two datasets and variables used for the analysis. Nn: number of nurses; Np: number of patients; PNR: patient-to-nurse ratio; Na: number of admissions; Nd: number of discharges; Nti: number of transfers in; Nto: number of transfers out; RN: registered nurse; LPN: licensed practical nurse.

The main activity data were drawn from 688,730 cases and 6834 nurses in 152 units. After the exclusion of outpatient units and noncare activities (whether direct or indirect), the activity data reflected 153,456 cases (153,456/688,730, 22.2%) and 5736 nurses (5736/6834, 83.9%) in 70 units (70/152, 46.1%). Of the remaining 5736 nurses, data from 4633 (80.8%) were usable for the final analyses. A number of nurses (1270) were excluded for specific days only, as they had recorded no working time data. Those exclusions correspond to 11,251 (1.5%) person-days. Another main reason for exclusion was that 1109 students and 227 external nurses did not have exact working hours. However, the data from both groups were usable for our descriptive analyses. Numerous students and external nurses became RNs over the 3 years of the study period. This largely explains why the final number of nurses was higher than 5736. Regarding the number of cases, a total of 128,484 (124,484/153,456, 83.7%) cases were used. Two main factors explain this reduction: outpatients (19,442/153,456, 12.7%) and healthy newborn babies (3779/153,456, 2.5%) from the Maternity & Gynecology department. Further details of the linking procedure are shown in Figure 1. For patient-to-nurse ratios and patient turnover analyses, we included 10 departments, including 70 inpatient units in which 4633 nurses (>22 million data points) provided care to 128,484 cases (>35 million data points).

### Descriptive Overview

The number of patient cases in the included units in each department over the study period (2015-2017) ranged from 5007 for Hematology & Oncology to 28,377 for Cardiology & Cardiovascular Surgery. In almost all departments, mean and median patient ages were >54 years. The exceptions were patients in Maternity & Gynecology*,* who had a mean age of 36.5 years (SD 15.4 years) and median age of 33 years (IQR 28-40 years), and in Pediatrics, who had mean and median ages of 3.8 years (SD 5 years) and 1 year (IQR 0-7 years), respectively. The Hematology & Oncology, Internal Medicine, and Orthopedics & Plastic Surgery departments had the highest median LOS, at 7 days (IQR 4-14 days), 6 days (IQR 3-10 days), and 5 days (IQR 3-9 days), respectively. Cardiology & Cardiovascular Surgery and Intensive Care had the lowest median LOS, at 2 days (IQR 1-7 days) and 2 days (IQR 2-3 days), respectively. The most common diagnoses were tumors, predominantly in the Hematology & Oncology department (4161/5007, 83.1%); circulatory system diseases, mainly in the Cardiology & Cardiovascular Surgery department (24,206/28,377, 85.3%); and traumatic injuries, poisonings, and other consequences of external causes, which were highest in the Orthopedics & Surgery department (5213/10,489, 49.7%). Further details are provided in Table 2.

**Table 2:** Descriptive overview of each department classified by the overall number of patients for 2015-2017.

Department	Number of patients	Number of units	Age (years), mean (SD)	Age (years), median (IQR^a^)	LOS^b^, median (IQR)	Patients/ day/unit, median (IQR)	Top 2 diagnoses, n/N (%)	
							First	Second
Cardiology & Cardiovascular Surgery	28,377	12	67.3 (14.4)	70 (59-78)	2 (2-5)	10 (7-12)	Circulatory system diseases, 24,206/28,377 (85.3)	Traumatic injuries, poisonings, and other consequences of external causes, 1220/28,377 (4.3)
Neurology, Neurosurgery, Otolaryngology, Head & Neck Surgery, & Ophthalmology	27,916	10	58.5 (18.5)	61 (47-73)	4 (3-6)	13 (11-17)	Circulatory system diseases, 6421/27,916 (23)	Nervous system diseases, 4327/27,916 (15.5)
Intensive Care	21,359	8	61.6 (16.6)	64 (52-74)	2 (2-3)	8 (7-10)	Circulatory system diseases, 8095/21,359 (37.9)	Tumors, 3503/21,359 (16.4)
Pediatrics	19,543	10	3.8 (5)	1 (0-7)	3 (2-7)	11 (8-14)	Some conditions whose origin is the perinatal period, 3987/19,543 (20.4)	Respiratory system diseases, 3479/19,543 (17.8)
Dermatology, Urology, Rheumatology, & Nephrology	16,381	7	59.6 (17.3)	62 (48-73)	4 (3-7)	12 (6-17)	Genitourinary system diseases, 5160/16,381 (31.5)	Tumors, 3473/16,381 (21.2)
Visceral Surgery and Medicine, Gastroenterology, Thoracic Surgery, & Pulmonology	14,250	5	58.2 (17.1)	61 (48-71)	4 (3-7)	17 (12-21)	Digestive system diseases, 5073/14,250 (35.6)	Tumors, 4190/14,250 (29.4)
Internal Medicine	12,506	6	66 (18.3)	70 (54-80)	6 (3-10)	15 (13-18)	Circulatory system diseases, 2389/12,506 (19.1)	Infectious and parasitic diseases, 1163/12,506 (9.3)
Maternity & Gynecology	11,894	3	36.5 (15.4)	33 (28-40)	4 (3-5)	18 (14-21)	Pregnancy, childbirth, and the puerperium, 7172/11,894 (60.3)	Tumors, 1998/11,894 (16.8)
Orthopedics & Plastic Surgery	10,489	5	52.9 (19.6)	54 (37-68)	5 (3-9)	14 (12-16)	Traumatic injuries, poisoning, and some other consequences of external causes, 5213/10,489 (49.7)	Diseases of the osteo-articular system, muscles and connective tissue, 3346/10,489 (31.9)
Hematology & Oncology	5007	4	59.2 (15.5)	61 (51-70)	7 (4-14)	11 (7-18)	Tumors, 4161/5007 (83.1)	Endocrine, nutritional, and metabolic diseases, 225/5007 (4.5)

^a^IQR: interquartile range.

^b^LOS: length of stay.

### Number of Patients

**Figure 2:**
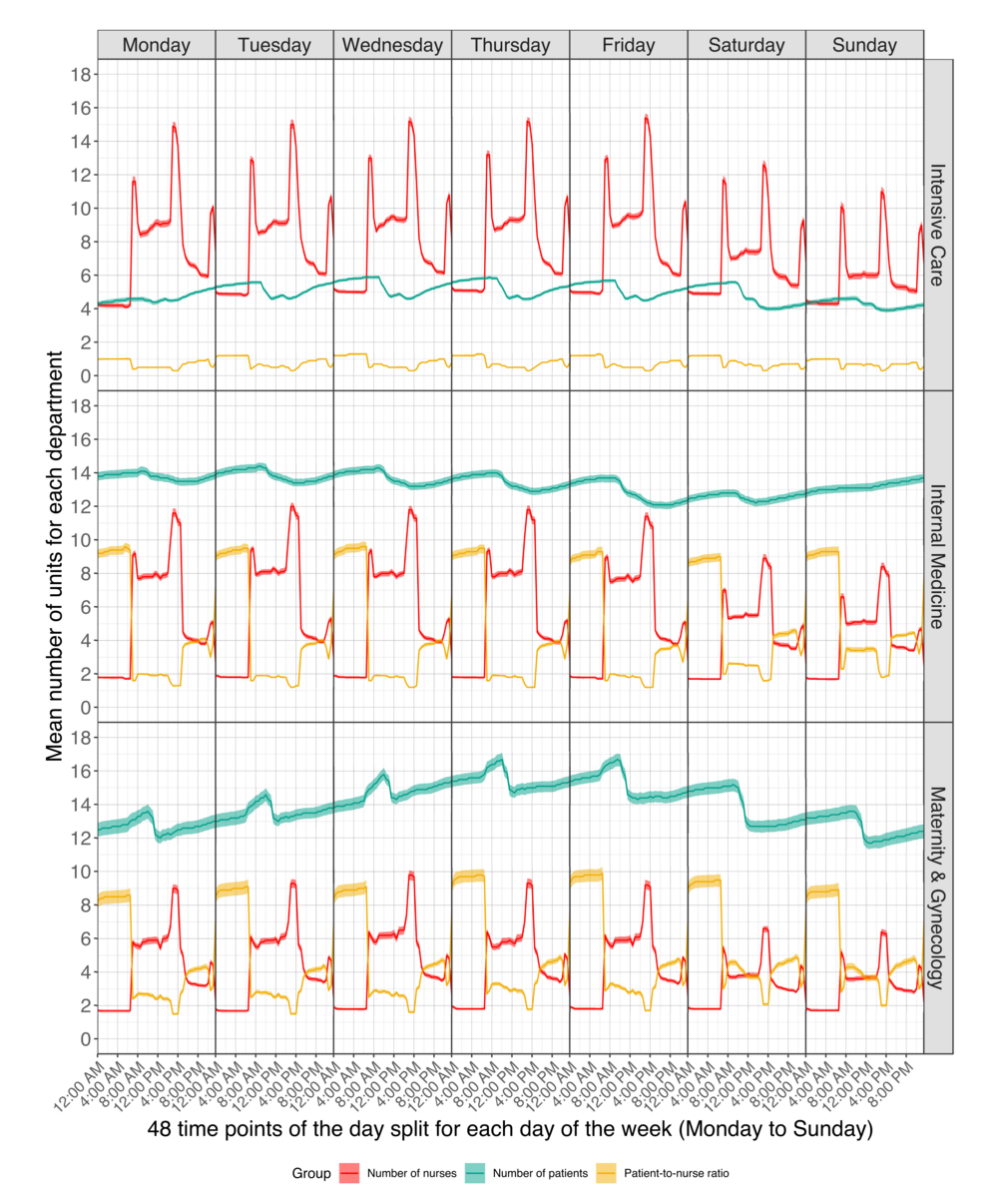
Plots of the number of patients, number of registered nurses, and patient-to-nurse ratio with the CIs.

Numbers of patients and nurses and patient-to-nurse ratios were plotted against the 48 data points per day for each day of the week, each of the 10 departments, and each of the 3 nurse groups. Considering the large number of plots this generated, we show only the 3 plots that show the key characteristic patterns: the RN group (group 1) for the Intensive Care, Maternity & Gynecology, and Internal Medicine departments (see Figure 2). All plots can be found in Multimedia Appendix 1 (group 1, RNs), Multimedia Appendix 2 (group 2, LPNs), and Multimedia Appendix 3 (group 3, others).

On the demand side, a number of broad patterns emerged, several of which occurred across departments. Demand fluctuated not only throughout the day, with various clear peaks, but also through the week, as shown for the Maternity & Gynecology department, where patient numbers peaked on Thursday and Friday. Overall, patient numbers increased for 6 departments (6/10, 60%) from Monday to Thursday or Friday, and patient numbers peaked daily between 8:00 am and 10:00 am, then either stabilized or decreased. On Friday mornings, patient numbers decreased in preparation for the weekend. The exception was in the Internal Medicine department, where the number of patients increased continuously from Friday evening until Monday morning (see Figure 2).

### Number of Nurses

From the care supply perspective, variation was far less pronounced than on the demand side. Three main variations were apparent. First, fewer nurses were present through the weekends and occasionally on Friday. Second, Sundays generally had fewer nurses than on Saturdays. Third, the numbers of nurses were highest in the morning, then dropped for the afternoon shift and again for the night shifts. These patterns where quite stable throughout the week. In 6 (6/10, 60%) of the departments, gaps of 1 or 2 nurses were clearly discernible between 11:00 am and 1:00 pm.

Except for 3 (30%) of the 10 departments with almost no staff on the weekends, 1-2 LPNs were mainly present between 06:00 am and 5:00 pm. The pattern for nurse group 3 (ie, others) was similar to that of LPNs, although generally with roughly 1 more care staff. All departments increased their staff by 1-3 nurses for all or several of the following times: 7:00 am to 8:00 am, 2:00 pm to 4:30 pm, and 10:00 pm to 12:00 am (see Figure 2). As mentioned, apart from daily information, no records of working time were available for either students or external nurses. For external nurses and students, the median daily number of nurses was 0, except in the Internal Medicine department*,* where there was a median of 1 student. The daily number of external nurses ranged from 0 to 9 and of students from 0 to 12. For both, the maxima occurred only once, on a Sunday, during the 3 years.

### Patient-to-nurse Ratio

**Figure 3:**
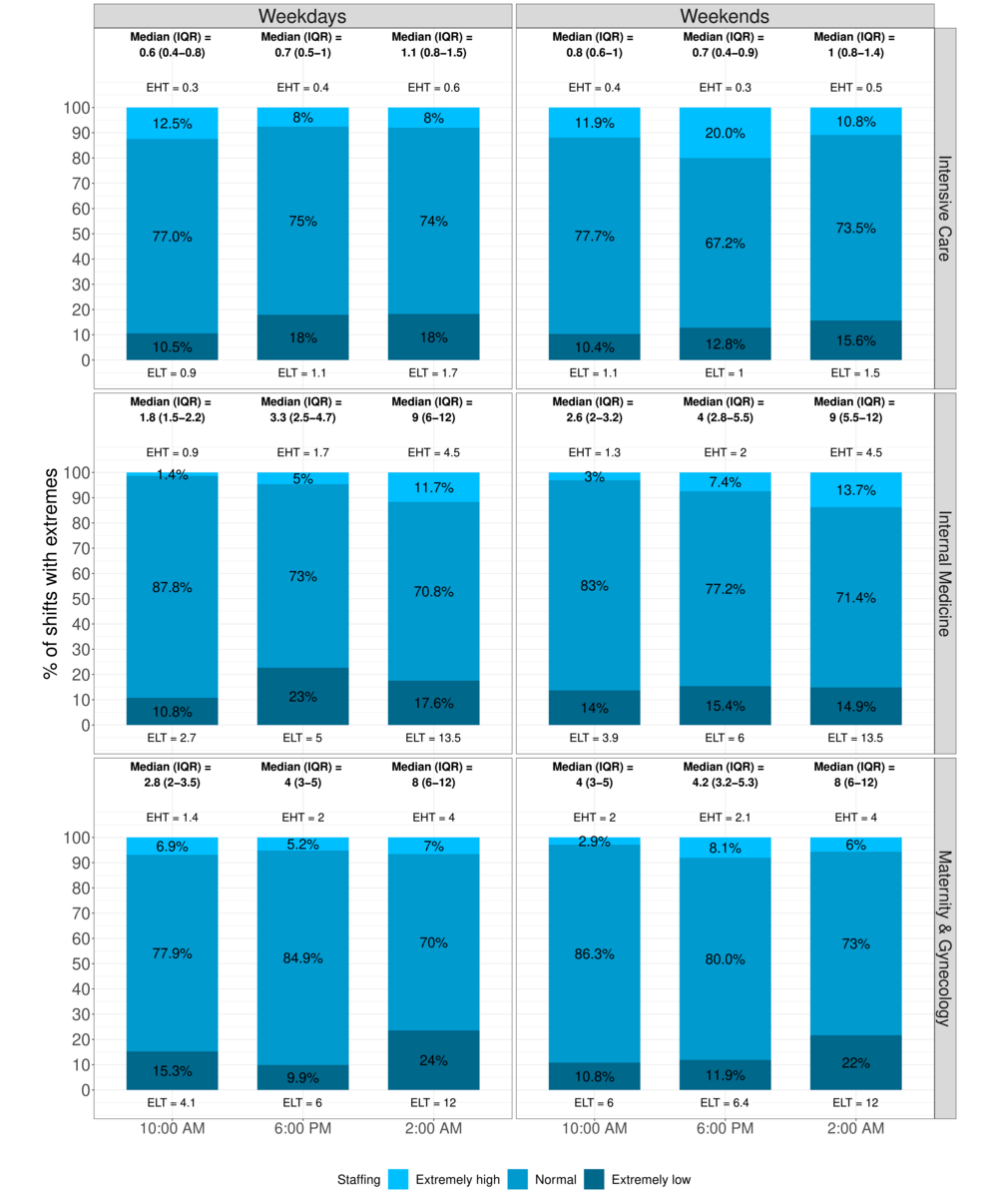
Median (interquartile range [IQR]) patient-to-registered nurse ratios for key time points, with the percentages of shifts with an extremely high threshold (EHT) or an extremely low threshold (ELT). Three departments are displayed split by weekdays and weekends.

Figure 2 shows the plots, and Figure 3 shows the median (IQR) of the patient-to-RN ratio for 3 key time points on weekdays and weekends. For RNs, the ratio was highest at night and lowest in the morning. During the night, the median ratio was 4-11 patients per RN, except in the Intensive Care department, which had a ratio of 1.1 patients per RN. In the morning, the ratio ranged from 0.6 (IQR 0.4-0.8) on weekdays to 0.8 (IQR 0.6-1) on weekends in the Intensive Care department and ranged from 2.8 (IQR 2-3.5) on weekdays to 4 (IQR 3-5) on weekends in the Maternity & Gynecology department. For LPNs, the median number was always 0 (IQR 0-0) at night, while the median number in the morning ranged from 3 to 8. For nurse group 3 (ie, others), a median number of 0 (IQR 0-0) staff members was generally present during the night shift. In the morning shifts, 7 departments’ (7/10, 70%) median patient-to-nurse ratios increased for group 3 (ie, others) from weekdays (4.3 to 8) to weekends (6 to 12). In the afternoon shifts, all medians decreased (see Multimedia Appendix 4).

### Extreme Mismatch Between Supply and Demand

Figure 3 shows not only the weekday and weekend medians (IQRs) of the patient-to-RN ratios for 3 key time points (namely 2 am, 10 am, and 6 pm) but also the threshold values and percentages of shifts with extreme supply-and-demand mismatches. Complete results can be found in Multimedia Appendix 4. For 7 departments (7/10, 70%), the percentages of shifts with extreme staffing increased from morning to night and from weekdays to weekends. For RNs, the lowest percentages of extreme understaffing and overstaffing happened on 2.5% and 0.1% of weekday and weekend mornings, respectively. For both extremely high and extremely low patient-to-nurse ratios, the highest incidence (around 30% of shifts) occurred in the evening and weekend nights. These ratios occurred in the Cardiology & Cardiovascular Surgery (lower); Dermatology, Urology, Rheumatology, & Nephrology (lower); and Hematology & Oncology (higher) departments. The same 3 departments had the lowest incidence of shifts with “normal” staffing levels (below 55%) for weekend nights. On the other hand, more than 80% of all shifts in the Orthopedics & Plastic Surgery department fell within “normal” staffing levels. For nurse groups 2 (LPNs) and 3 (others), the incidence of extreme staffing ranged from very high (49.5%) to very low (1.5%) to none (0%). Possibly because of these groups’ low nurse numbers, no clear patterns were apparent.

### Patient Turnover

**Figure 4:**
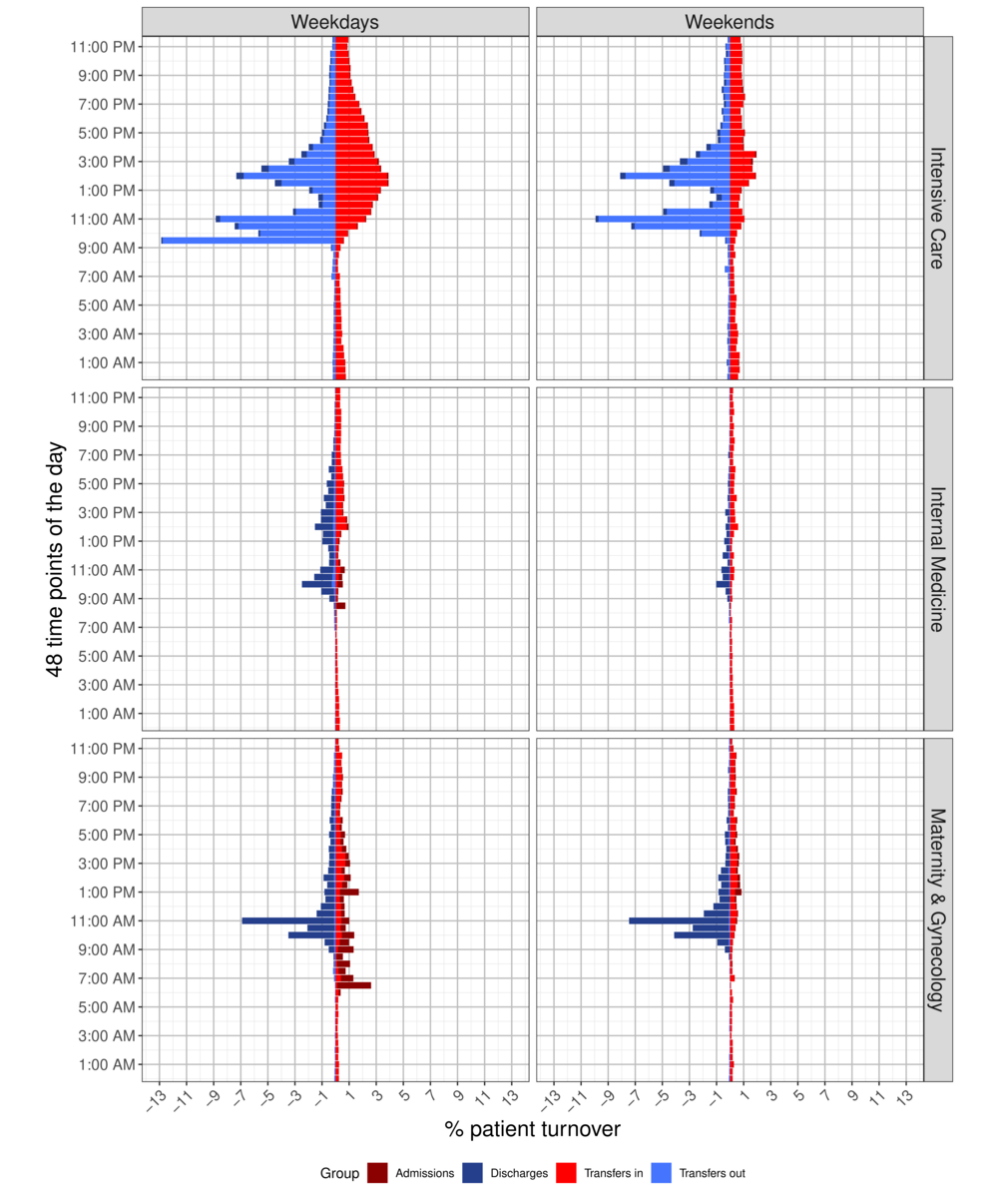
Percentages of patient turnover for the 48 data points by weekdays and weekends.

Similar to the patient-to-nurse ratio, bar charts for patient turnover are displayed for only the Intensive Care, Internal Medicine, and Maternity & Gynecology departments (see Figure 4). Bar charts for all 10 departments are displayed in Multimedia Appendix 5. All departments showed reductions in patient turnover during the weekends. Entries (admissions and transfers in) and exits (discharges and transfers out) of patients occurred at very similar times for all departments: 09:00 am to 11:00 am and 1:00 pm to 3:00 pm. The Intensive Care department had the highest percentage of transfers (peaking at almost 13% at 9:30 am). As shown in Figure 3, the numbers of extreme staffing mismatches also fluctuated as a result of the number of either nurses or patients present. Figure 4 shows the variation in the patterns throughout the day that influenced the patient-to-nurse ratio. For example, if on a given day a peak of discharges occurs at 10:00 am with no or few admissions or transfers in, the patient-to-nurse ratio will decrease, potentially leading to extreme overstaffing. The same is true for the inverse. A peak in admissions or transfers in can increase the patient-to-nurse ratio, leading to extreme understaffing.

## Discussion

### Main Results

For the first time, we longitudinally analyzed demand for and supply of care at 30-minute intervals for 24 hours a day over a period of 3 years in a large university hospital. Data from the nurse staffing system (tacs) and medical discharge records were used to explore patient-to-nurse ratios and patient turnover. The 10 departments belonged to the Inselspital (Bern University Hospital) and varied by purpose, size, number of units, and patient population.

From the demand side, continuous turnover meant patient numbers fluctuated across each day and varied across units. Less variation was seen in the supply side, where the change in the number of nurses occurred mainly for each shift (night, morning, evening). RNs accounted for roughly three-quarters of the nurse workforce, making them the largest staff subgroup involved with patient care. The remaining quarter was comprised of others (including unlicensed personnel and administrative staff) and LPNs. These smaller groups were mainly present during day shifts on weekdays.

Simultaneous longitudinal data on patient and nurse numbers allowed us to determine which had the greater influence on patient-to-nurse ratios (ie, the effects the variations in the numbers of nurses and patients had on nurse workload). Most published studies have shown that during weekends, patient-to-nurse ratios tend to increase [6,7]. We confirmed this observation, not only because the nursing staff was reduced over weekends but also because in many departments (eg, Internal Medicine), patient numbers tended to increase on Saturday and Sunday. In fact, we found that the supply side remained quite constant; it was mainly the demand side that drove patient-to-nurse ratios.

The ramifications of a demand-driven workload are particularly clear regarding weekend staff planning. Reducing staffing for reduced weekend demand might be justified on Friday nights, as a surge in discharges Thursday and Friday decreases patient numbers. However, patient numbers increased in several departments over the weekends, as patient entries outnumbered exits.

To set the reported patient-to-nurse ratios in context, the European cross-sectional study RN4CAST, which was conducted in 2009-2011 with 35 Swiss acute care hospitals, reported an average of 7.9 patients per nurse in medical and surgical units [37,38]. However, the reported ratios were aggregated at the hospital level, ignoring the ratios per shift. In 2015, the Swiss cross-sectional Match^RN^ study followed up the same hospitals that participated in RN4CAST [39]. The overall average patient-to-nurse ratio of the 23 participating hospitals was 7.8, and the shift averages were 5.9 for the morning shift, 7.3 for the evening shift, and 14.2 for the night shift [40,41]. Both cross-sectional studies surveyed only RNs. From our study, the median patient-to-nurse ratio for RNs was 2-3 for the morning shift, 3-5 for the evening shift, and 4-10 for the night shift, excluding the Pediatrics, Maternity & Gynecology, and Intensive Care departments. The ratios are difficult to compare with the overall averages from the RN4CAST and Match^RN^ studies. At the shift level, our patient-to-nurse ratios were lower than the Match^RN^ ratios, suggesting an above-average staffed hospital. High patient-to-nurse ratios have been associated with worse patient outcomes; however, due to conflicting results, the relationship remains unclear [42,43]. One main reason for the lack of conclusive evidence is researchers’ tendency to seek associations between mean patient-to-nurse ratios and patient outcomes [44]. However, this means obscuring sharp changes in supply and demand, thereby concealing periods when staffing levels are low. As no consensus exists concerning the definition of an extreme staffing situation, we chose arbitrary cut-offs of double or half of the median work per nurse to define extremely high or low patient-to-nurse ratios, respectively. These cut-offs showed that our extreme thresholds were commonly crossed during certain shifts and in certain departments.

This observation underscores the potential volatility of nurse workload, even over a single shift, and the need for longitudinal approaches to staffing research to help identify and counteract this volatility [45]. The distribution of extreme shifts also indicated that individual departments (eg, the Orthopedics & Surgery department, with more than 80% of units and shifts staffed within the “normal” range) can maintain their patient-to-nurse ratio quite effectively. Identifying the most meaningful thresholds to define extreme staffing will require further research.

As illustrated in Figure 4, while patient turnover was continuous, it was concentrated at various times throughout the day. Consistent with previous findings, entries and exits were both rare during the night [46,47]. Moving patients to the units where they can receive the most appropriate care is essential for their recovery. Also, as demand is independent of available resources, patient turnover occurs when units are short on either staff or beds. For the former, the unit is closed, and the patient is moved to a similar unit; for the latter, the patient is placed in an intermediate unit until a bed becomes available [48]. However, both cases lead to increased administrative work, and even where beds are available and staff sufficient, transfers, admissions, and discharges all entail higher volumes of administrative requirements and patient care needs. Therefore, our analyses confirmed that patient turnover is one factor for nursing workload [5,47,49].

The impact of turnover can be greater when entries and exits occur at the same time, as illustrated in the first row of Figure 4 for the Intensive Care department. Between 1.6% and 32.3% of nursing time is spent on patient turnover-related activities [50,51]. Associations between patient turnover and nursing care quality have been documented, where higher turnover led to higher nursing workload, possibly compromising nursing care quality [13,38,52]. High patient turnover is associated with more adverse events, including mortality [53], nosocomial infections [54], and medication errors [52] as well as more readmissions [55,56]. The current approach is somewhat unrealistic. Where each patient case or event is rooted in a unique context, much of the current literature treats all patient turnover and patients as the same [47,57]. In contrast, Tierney et al [58] used weighted patient acuity and patient turnover variables to account for intercase variation. Studies also showed that death was a more likely outcome in contexts featuring high patient-to-nurse ratios and patient turnover [13,59,60].

### Potential Implications

Because of the granularity of the analysis, patient-to-nurse ratios were analyzed with patient turnover, as even in cases where the patient-to-nurse ratio might appear normal, both patient entries and exits increase nurse workload. During periods of high turnover, the time available for patient care can be severely reduced. Hospitals or departments that fail to account for this additional burden commonly operate with suboptimal nursing staff levels [51-53].

Previous research has suggested a relationship between higher patient-to-nurse ratios and worse patient outcomes [61-66]. Mandatory minimum patient-to-nurse ratios are often suggested as an approach to ensure safe staffing levels. As the nursing supply is quite constant at the level of individual shifts, the question may arise as to whether that supply can realistically be changed in response to midshift fluctuations in demand. The patient turnover variability illustrated in this study showed that where entries matched exits, patient numbers remained reasonably constant and where imbalances occurred between entries and exits, patient numbers fluctuated.

However, neither of these cases adequately reflects nurse workload. In the first, even while a balanced turnover resulted in a constant patient-to-nurse ratio, if both sides increased, the additional work required for each incoming and outgoing case would represent a considerable burden. In the second, records of patient numbers alone give no indication as to whether the supply of nurses was adjusted accordingly. These two examples illustrate the necessity of considering both supply and demand data for staffing purposes.

Certain patterns were clearly associated with routines that applied to specific days and times of the week. Defining and clarifying those periods for each unit would help improve assessment of staffing levels. Given that some hospital departments do not operate on the weekends, further detailed analysis of weekend work for nurses is needed to determine how the workload increases. Current research on hospital staffing is predominantly based on cross-sectional data, which cannot show fluctuations in patient turnover [67]. Lacking longitudinal data, it is extremely difficult to match the rather constant nurse supply to the varying patterns in patient demand.

To our knowledge, only one previous study examined the longitudinal associations between nurse staffing and patient turnover. Its findings indicated large variations in patient turnover [68]. As the demand side is quite volatile, to anticipate when the nurse supply should be changed to match changes in workload, it is important to identify the times of day, days of the week, and even months during which specific entry and exit patterns occur. Armed with this information, staffing levels might differ across not only units and hospitals but also countries. Thus, unit-level analysis offers the best chance to detect patterns of supply and demand. Identifying the complex relationships involved and then building more efficient predictive models that capture all meaningful variations will require further studies examining longitudinal nurse and patient data.

### Limitations

Certain limitations were encountered during our analyses. One of these concerned the tacs nurse staffing system, as this was the first time that routine data were used for research and linking purposes. During the process, we found that a small minority of nurses (~ 1%) were not using the system consistently. Also, due to issues with merging data, a number of nurses and patients were excluded from the analysis. To maximize the data quality for this and future studies, these issues were discussed with the nurse staffing system software developers.

Also, while we selected persons providing direct or indirect care for the analysis, it was impossible to know whether those people also performed tasks not associated with patient care, such as organizational tasks or teaching. Patient-to-nurse ratios were calculated for all persons present in each unit studied. Although we measured variation in nurses’ workload with a high level of granularity, the significance of the short peaks in demand relative to the supply over short periods is hard to judge because nurses’ work involves multitasking and they can prioritize urgent tasks and delay others without necessarily harming patients [69]. Minutes of care were also available from the data; however, due to data quality concerns regarding the time allocated to each patient, these data were excluded, and metadata were included in their place. This may have solved the patient care time data limitation by providing the exact time invested for each patient during working hours.

Further, the results were limited by the absence of accurate working time data for external nurses and students. Even if these groups had a daily median presence of 0, their assistance might have been crucial when they were present, as for night shift support. This type of task shifting between individuals and departments to compensate for staffing shortfalls is a key tool to handle demand and avoid gaps in supply but was not recorded in the available data.

Our study looked only at nurses, but the hospital environment is multidisciplinary. All health care providers play an important role, and their collaboration is crucial for patients [70]. For example, studies showed a positive impact on patients’ outcomes by incorporating or improving nurse-physician or pharmacist-physician collaborative practices [71-73].

Finally, the study was undertaken in a single hospital, and we explored many sources of variation, but not patient acuity and severity. Nursing workload depends on not only the amount of direct and indirect care, patient turnover, and patient-to-nurse ratio but also patient acuity and severity [74,75]. Further investigation is thus needed.

### Conclusions

To our knowledge, this is the first detailed study to use data on patient-to-nurse ratios and patient turnover in time increments as low as 30 minutes. The goal was to illustrate fluctuations in these two variables between and within departments and days of the week. The choice of 30 minutes was subjective and based on available computational resources. While the literature includes references to the fluctuations we observed, no study to date has illustrated those fluctuations in such fine detail. The key driver for care was clearly patient demand, which showed high variability even during individual shifts. This volatility challenges health care suppliers to provide safe and reliable care when demand is high while avoiding overstaffing when it is low. Detecting patterns of variation will help optimize staffing. This descriptive analysis was a first step towards detecting fluid variables to be considered in developing a predictive model on which to base health care staff planning, possibly including the introduction of innovative working/shift schemes in this sensitive sector.

## Appendix

Multimedia Appendix 1 Number of patients and nurses with patient-to-nurse ratios plotted (with confidence intervals) for RN group. The x-axis shows the 48 time points of the day split for each day of the week (Monday to Sunday); the y-axis represents the mean number of units for each of the ten departments.

Multimedia Appendix 2 Number of patients and nurses with patient-to-nurse ratios plotted (with confidence intervals) for LPN group. The x-axis shows the 48 time points of the day split for each day of the week (Monday to Sunday); the y-axis represents the mean number of units for each of the ten departments.

Multimedia Appendix 3 Number of patients and nurses, with patient-to-nurse ratios plotted (with confidence intervals) for Others group. The x-axis shows the 48 time points of the day split for each day of the week (Monday to Sunday); the y-axis represents the mean number of units for each of the ten departments.

Multimedia Appendix 4 Median of patient−to−nurse ratio for key time points, split by weekdays/weekends for each group of nurses, together with percentages of shifts with extreme patient−to−nurse ratios.

Multimedia Appendix 5 Patient turnover in percentages for the 48 data points, split for weekdays and weekends.

## Abbreviations

IQR: interquartile range.
LOS: length of stay.
LPNs: licensed practical nurses.
RNs: registered nurses.

## References

[1] Aiken LH, Clarke SP, Sloane DM, Sochalski J, Silber JH (2002). Hospital nurse staffing and patient mortality, nurse burnout, and job dissatisfaction. JAMA.

[2] Mark BA, Hughes LC, Belyea M, Chang Y, Hofmann D, Jones CB, Bacon CT (2007). Does safety climate moderate the influence of staffing adequacy and work conditions on nurse injuries?. J Safety Res.

[3] Manojlovich M, Sidani S, Covell CL, Antonakos CL (2011). Nurse dose: linking staffing variables to adverse patient outcomes. Nurs Res.

[4] Fasoli DR, Haddock KS (2010). Results of an integrative review of patient classification systems. Annu Rev Nurs Res.

[5] Swiger PA, Vance DE, Patrician PA (2016). Nursing workload in the acute-care setting: A concept analysis of nursing workload. Nurs Outlook.

[6] Palese A, Petean M, Cerne D (2014). Unexpected deaths in medical wards during night shifts: a narrative analysis of nursing experiences. J Clin Nurs.

[7] Patrician PA, Loan L, McCarthy M, Fridman M, Donaldson N, Bingham M, Brosch LR (2011). The association of shift-level nurse staffing with adverse patient events. J Nurs Adm.

[8] Aspden P, Wolcott J, Bootman J, Cronenwett L (2007). Preventing medication errors: quality chasm series. Committee on Identifying and Preventing Medication Errors.

[9] Blay N, Duffield CM, Gallagher R (2012). Patient transfers in Australia: implications for nursing workload and patient outcomes. J Nurs Manag.

[10] Blay N, Roche MA, Duffield C, Gallagher R (2017). Intrahospital transfers and the impact on nursing workload. J Clin Nurs.

[11] Duffield C, Diers D, Aisbett C, Roche M (2009). Churn: patient turnover and case mix. Nurs Econ.

[12] McGillis Hall L, Kiesners D (2005). A narrative approach to understanding the nursing work environment in Canada. Soc Sci Med.

[13] Needleman J, Buerhaus P, Pankratz VS, Leibson CL, Stevens SR, Harris M (2011). Nurse staffing and inpatient hospital mortality. N Engl J Med.

[14] Griffiths P, Maruotti A, Recio Saucedo A, Redfern OC, Ball JE, Briggs J, Dall'Ora C, Schmidt PE, Smith GB, Missed Care Study Group (2019). Nurse staffing, nursing assistants and hospital mortality: retrospective longitudinal cohort study. BMJ Qual Saf.

[15] Huber E, Kleinknecht-Dolf M, Müller Marianne, Kugler C, Spirig R (2017). Mixed-method research protocol: defining and operationalizing patient-related complexity of nursing care in acute care hospitals. J Adv Nurs.

[16] Welton JM (2017). Measuring Patient Acuity: Implications for Nurse Staffing and Assignment. J Nurs Adm.

[17] Halm M, Peterson M, Kandels M, Sabo J, Blalock M, Braden R, Gryczman A, Krisko-Hagel K, Larson D, Lemay D, Sisler B, Strom L, Topham D (2005). Hospital nurse staffing and patient mortality, emotional exhaustion, and job dissatisfaction. Clin Nurse Spec.

[18] Titler M, Dochterman J, Xie X, Kanak M, Fei Q, Picone DM, Shever L (2006). Nursing interventions and other factors associated with discharge disposition in older patients after hip fractures. Nurs Res.

[19] Weiss ME, Piacentine LB, Lokken L, Ancona J, Archer J, Gresser S, Holmes SB, Toman S, Toy A, Vega-Stromberg T (2007). Perceived readiness for hospital discharge in adult medical-surgical patients. Clin Nurse Spec.

[20] Hripcsak G, Friedman C, Alderson PO, DuMouchel W, Johnson SB, Clayton PD (1995). Unlocking clinical data from narrative reports: a study of natural language processing. Ann Intern Med.

[21] Diya Luwis, Van den Heede Koen, Sermeus Walter, Lesaffre Emmanuel (2012). The relationship between in-hospital mortality, readmission into the intensive care nursing unit and/or operating theatre and nurse staffing levels. J Adv Nurs.

[22] Griffiths P, Ball J, Drennan J, Dall'Ora C, Jones J, Maruotti A, Pope C, Recio Saucedo A, Simon M (2016). Nurse staffing and patient outcomes: Strengths and limitations of the evidence to inform policy and practice. A review and discussion paper based on evidence reviewed for the National Institute for Health and Care Excellence Safe Staffing guideline development. Int J Nurs Stud.

[23] Welton JM (2016). Nurse staffing and patient outcomes: Are we asking the right research question?. Int J Nurs Stud.

[24] Clarke S, Donaldson N, Hughes RG (2008). Nurse staffing and patient care quality and safety. Patient safety and quality: An evidence-based handbook for nurses.

[25] Yakusheva O, Lindrooth R, Weiss M (2014). Economic evaluation of the 80% baccalaureate nurse workforce recommendation: a patient-level analysis. Med Care.

[26] Insel Gruppe AG.

[27] R Core Team (2013). Development.

[28] Henry L, Wickham H (2018). R package version 0.2.

[29] Wickham H, François R, Henry L, Müller K (2018). R package version 0.7.6.

[30] Wickham H (2019). RDocumentation.

[31] Dowle M, Srinivasan A (2017). R package version 1.10.

[32] Grolemund G, Wickham H (2011). Dates and Times Made Easy with {lubridate}. J Stat Soft.

[33] James D, Hornik K (2018). R package version 23.

[34] Thoen E (2018). R package version 0.4.1.

[35] Wickham H (2016). ggplot2: elegant graphics for data analysis.

[36] Wickham H (2018). R package version 1.0.0.

[37] Sermeus W, Aiken LH, Van den Heede K, Rafferty AM, Griffiths P, Moreno-Casbas MT, Busse R, Lindqvist R, Scott AP, Bruyneel L, Brzostek T, Kinnunen J, Schubert M, Schoonhoven L, Zikos D, RN4CAST consortium (2011). Nurse forecasting in Europe (RN4CAST): Rationale, design and methodology. BMC Nurs.

[38] Aiken LH, Sloane DM, Bruyneel L, Van den Heede K, Sermeus W, RN4CAST Consortium (2013). Nurses' reports of working conditions and hospital quality of care in 12 countries in Europe. Int J Nurs Stud.

[39] Bachnick S, Ausserhofer D, Januel J, Schubert M, Schwendimann R, De Geest S, Simon M (2017). Matching Registered Nurse services with changing care demands (Match ): study protocol of a natural experiment multi-centre study. J Adv Nurs.

[40] Bachnick S, Ausserhofer D, Baernholdt M, Simon M, Match RN study group (2018). Patient-centered care, nurse work environment and implicit rationing of nursing care in Swiss acute care hospitals: A cross-sectional multi-center study. Int J Nurs Stud.

[41] Dhaini SR, Denhaerynck K, Bachnick S, Schwendimann R, Schubert M, De Geest S, Simon M, Match RN study group (2018). Work schedule flexibility is associated with emotional exhaustion among registered nurses in Swiss hospitals: A cross-sectional study. Int J Nurs Stud.

[42] Sales A, Sharp N, Li Y, Lowy E, Greiner G, Liu C, Alt-White A, Rick C, Sochalski J, Mitchell PH, Rosenthal G, Stetler C, Cournoyer P, Needleman J (2008). The association between nursing factors and patient mortality in the Veterans Health Administration: the view from the nursing unit level. Med Care.

[43] Numata Y, Schulzer M, van der Wal R, Globerman J, Semeniuk P, Balka E, Fitzgerald JM (2006). Nurse staffing levels and hospital mortality in critical care settings: literature review and meta-analysis. J Adv Nurs.

[44] Lee A, Cheung YSL, Joynt GM, Leung CCH, Wong W, Gomersall CD (2017). Are high nurse workload/staffing ratios associated with decreased survival in critically ill patients? A cohort study. Ann Intensive Care.

[45] Schubert M, Glass TR, Clarke SP, Aiken LH, Schaffert-Witvliet B, Sloane DM, De Geest S (2008). Rationing of nursing care and its relationship to patient outcomes: the Swiss extension of the International Hospital Outcomes Study. Int J Qual Health Care.

[46] Baernholdt M, Cox K, Scully K (2010). Using clinical data to capture nurse workload: implications for staffing and safety. Comput Inform Nurs.

[47] Jennings BM, Sandelowski M, Higgins MK (2013). Turning over patient turnover: an ethnographic study of admissions, discharges, and transfers. Res Nurs Health.

[48] VanFosson CA, Yoder LH, Jones TL (2017). Patient Turnover: A Concept Analysis. ANS Adv Nurs Sci.

[49] Myny D, Van Hecke A, De Bacquer D, Verhaeghe S, Gobert M, Defloor T, Van Goubergen D (2012). Determining a set of measurable and relevant factors affecting nursing workload in the acute care hospital setting: a cross-sectional study. Int J Nurs Stud.

[50] Abbey M, Chaboyer W, Mitchell M (2012). Understanding the work of intensive care nurses: a time and motion study. Aust Crit Care.

[51] Cornell P, Herrin-Griffith D, Keim C, Petschonek S, Sanders AM, D'Mello S, Golden TW, Shepherd G (2010). Transforming nursing workflow, part 1: the chaotic nature of nurse activities. J Nurs Adm.

[52] Duffield C, Diers D, O'Brien-Pallas L, Aisbett C, Roche M, King M, Aisbett K (2011). Nursing staffing, nursing workload, the work environment and patient outcomes. Appl Nurs Res.

[53] Wagner J, Gabler NB, Ratcliffe SJ, Brown SES, Strom BL, Halpern SD (2013). Outcomes among patients discharged from busy intensive care units. Ann Intern Med.

[54] Cunningham JB, Kernohan WG, Rush T (2006). Bed occupancy, turnover intervals and MRSA rates in English hospitals. Br J Nurs.

[55] Heggestad T (2001). Operating conditions of psychiatric hospitals and early readmission--effects of high patient turnover. Acta Psychiatr Scand.

[56] Wagner C, Budreau G, Everett LQ (2005). Analyzing fluctuating unit census for timely staffing intervention. Nurs Econ.

[57] Fieldston ES, Ragavan M, Jayaraman B, Allebach K, Pati S, Metlay JP (2011). Scheduled admissions and high occupancy at a children's hospital. J Hosp Med.

[58] Tierney SJ, Seymour-Route P, Crawford S (2013). Weighted staffing plans for better prediction of staffing needs. J Nurs Adm.

[59] Neuraz A, Guérin Claude, Payet C, Polazzi S, Aubrun F, Dailler F, Lehot J, Piriou V, Neidecker J, Rimmelé Thomas, Schott A, Duclos A (2015). Patient Mortality Is Associated With Staff Resources and Workload in the ICU: A Multicenter Observational Study. Crit Care Med.

[60] Griffiths P, Ball J, Bloor K, Böhning D, Briggs J, Dall'Ora C, De IA, Jones J, Kovacs C, Maruotti A, Meredith P, Prytherch D, Saucedo A, Redfern O, Schmidt P, Sinden N, Smith G (2018). Nurse staffing levels, missed vital signs observations and mortality in hospital wards: retrospective longitudinal observational study. Health Services and Delivery Research.

[61] Needleman J, Buerhaus P, Mattke S, Stewart M, Zelevinsky K (2002). Nurse-staffing levels and the quality of care in hospitals. N Engl J Med.

[62] Blegen MA, Goode CJ, Reed L (1998). Nurse staffing and patient outcomes. Nurs Res.

[63] Kovner C, Gergen P J (1998). Nurse staffing levels and adverse events following surgery in U.S. hospitals. Image J Nurs Sch.

[64] Rafferty AM, Clarke SP, Coles J, Ball J, James P, McKee M, Aiken LH (2007). Outcomes of variation in hospital nurse staffing in English hospitals: cross-sectional analysis of survey data and discharge records. Int J Nurs Stud.

[65] McCloskey JM (1998). Nurse staffing and patient outcomes. Nurs Outlook.

[66] Weissman JS, Rothschild JM, Bendavid E, Sprivulis P, Cook EF, Evans RS, Kaganova Y, Bender M, David-Kasdan J, Haug P, Lloyd J, Selbovitz LG, Murff HJ, Bates DW (2007). Hospital workload and adverse events. Med Care.

[67] Driscoll A, Grant MJ, Carroll D, Dalton S, Deaton C, Jones I, Lehwaldt D, McKee G, Munyombwe T, Astin F (2018). The effect of nurse-to-patient ratios on nurse-sensitive patient outcomes in acute specialist units: a systematic review and meta-analysis. European Journal of Cardiovascular Nursing.

[68] He J, Staggs VS, Bergquist-Beringer S, Dunton N (2016). Nurse staffing and patient outcomes: a longitudinal study on trend and seasonality. BMC Nurs.

[69] Patterson ES, Ebright PR, Saleem JJ (2011). Investigating stacking: How do registered nurses prioritize their activities in real-time?. International Journal of Industrial Ergonomics.

[70] Morley L, Cashell A (2017). Collaboration in Health Care. J Med Imaging Radiat Sci.

[71] Brown SS, Lindell DF, Dolansky MA, Garber JS (2015). Nurses' professional values and attitudes toward collaboration with physicians. Nurs Ethics.

[72] Matzke GR, Moczygemba LR, Williams KJ, Czar MJ, Lee WT (2018). Impact of a pharmacist-physician collaborative care model on patient outcomes and health services utilization. Am J Health Syst Pharm.

[73] Sabone M, Mazonde P, Cainelli F, Maitshoko M, Joseph R, Shayo J, Morris B, Muecke M, Wall BM, Hoke L, Peng L, Mooney-Doyle K, Ulrich CM (2020). Everyday ethical challenges of nurse-physician collaboration. Nurs Ethics.

[74] Acar I, Butt SE (2016). Modeling nurse-patient assignments considering patient acuity and travel distance metrics. J Biomed Inform.

[75] Alghamdi MG (2016). Nursing workload: a concept analysis. J Nurs Manag.

