# Supplementary material for: Longitudinal Study of the Variation in Patient Turnover and Patient-to-Nurse Ratio: Descriptive Analysis of a Swiss University Hospital
Source: J Med Internet Res. 2020 Apr 2;22(4):e15554. doi: 10.2196/15554 (PMC7163415; doi:10.2196/15554)

Internal Medicine − licensed practical nurses

x−axis showed the 48 time points of the day split for each day of the week (Monday to Sunday), where y−axis represented the mean number of units with confidence interval

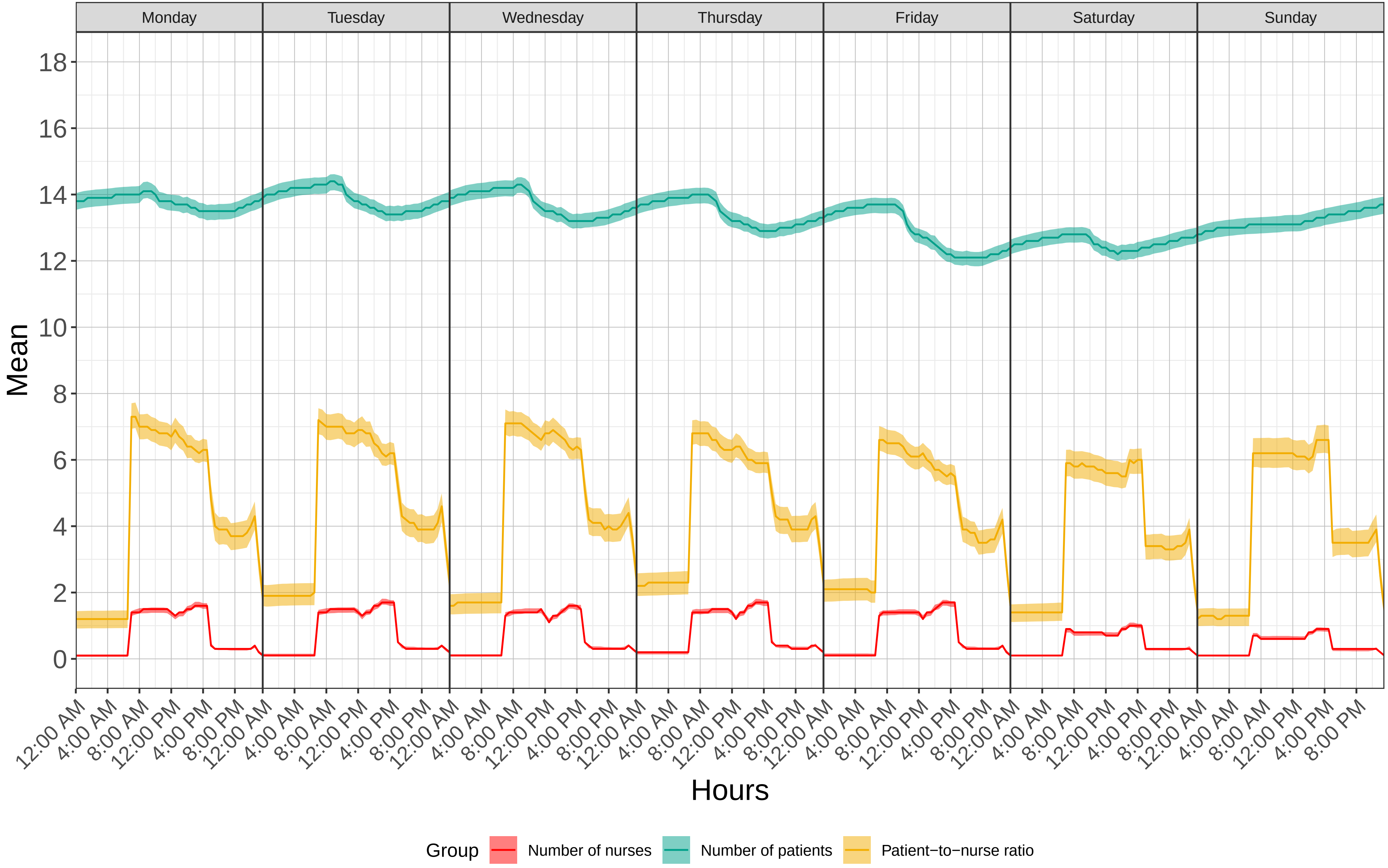

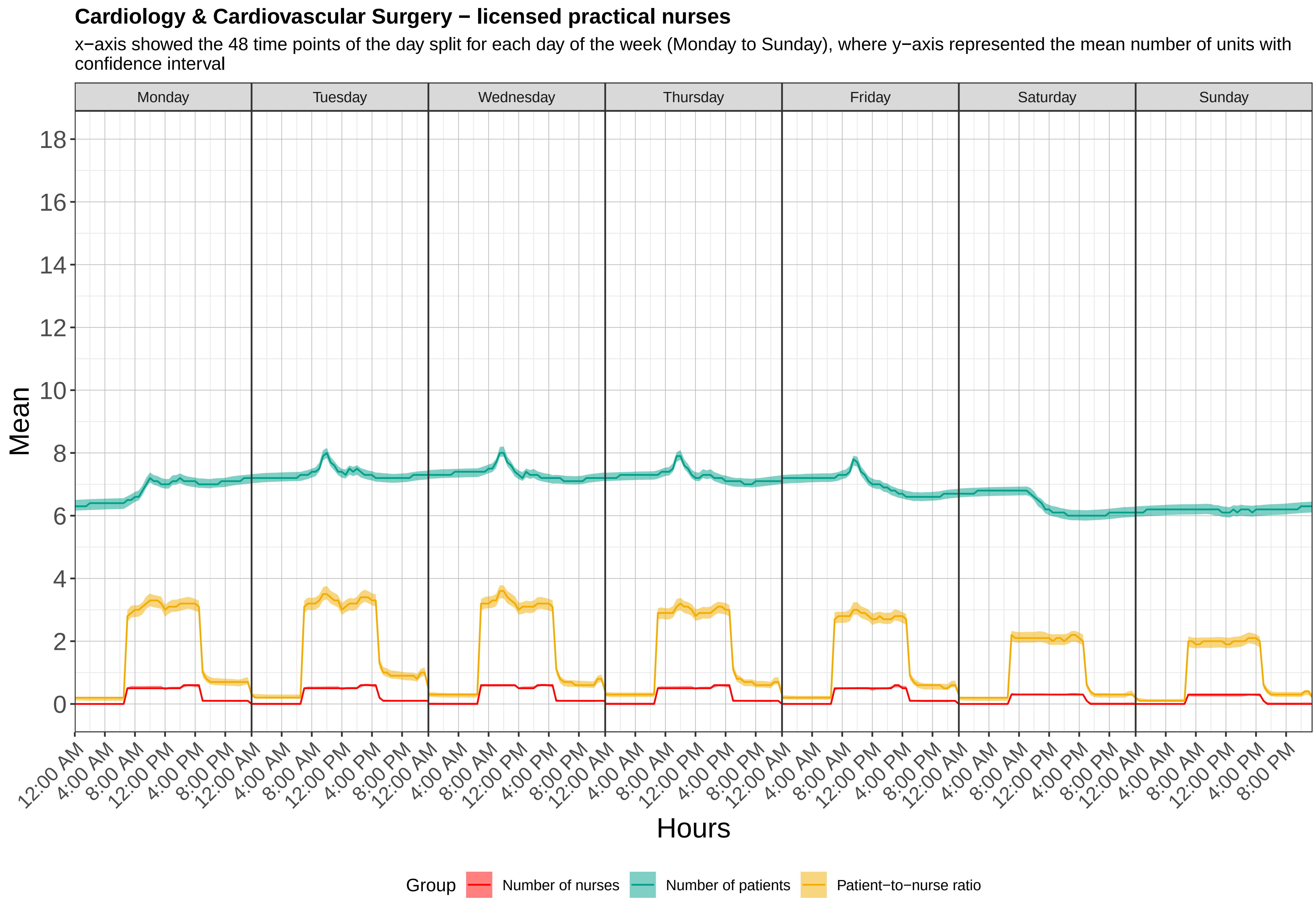
Cardiology & Cardiovascular Surgery − licensed practical nurses
x−axis showed the 48 time points of the day split for each day of the week (Monday to Sunday), where y−axis represented the mean number of units with confidence interval
Monday
Tuesday
Wednesday
Thursday
Friday
Saturday
Sunday
Mean
0
2
4
6
8
10
12
14
16
18
12:00 AM
4:00 AM
8:00 AM
12:00 PM
4:00 PM
8:00 PM
Hours
Group
Number of nurses
Number of patients
Patient−to−nurse ratio

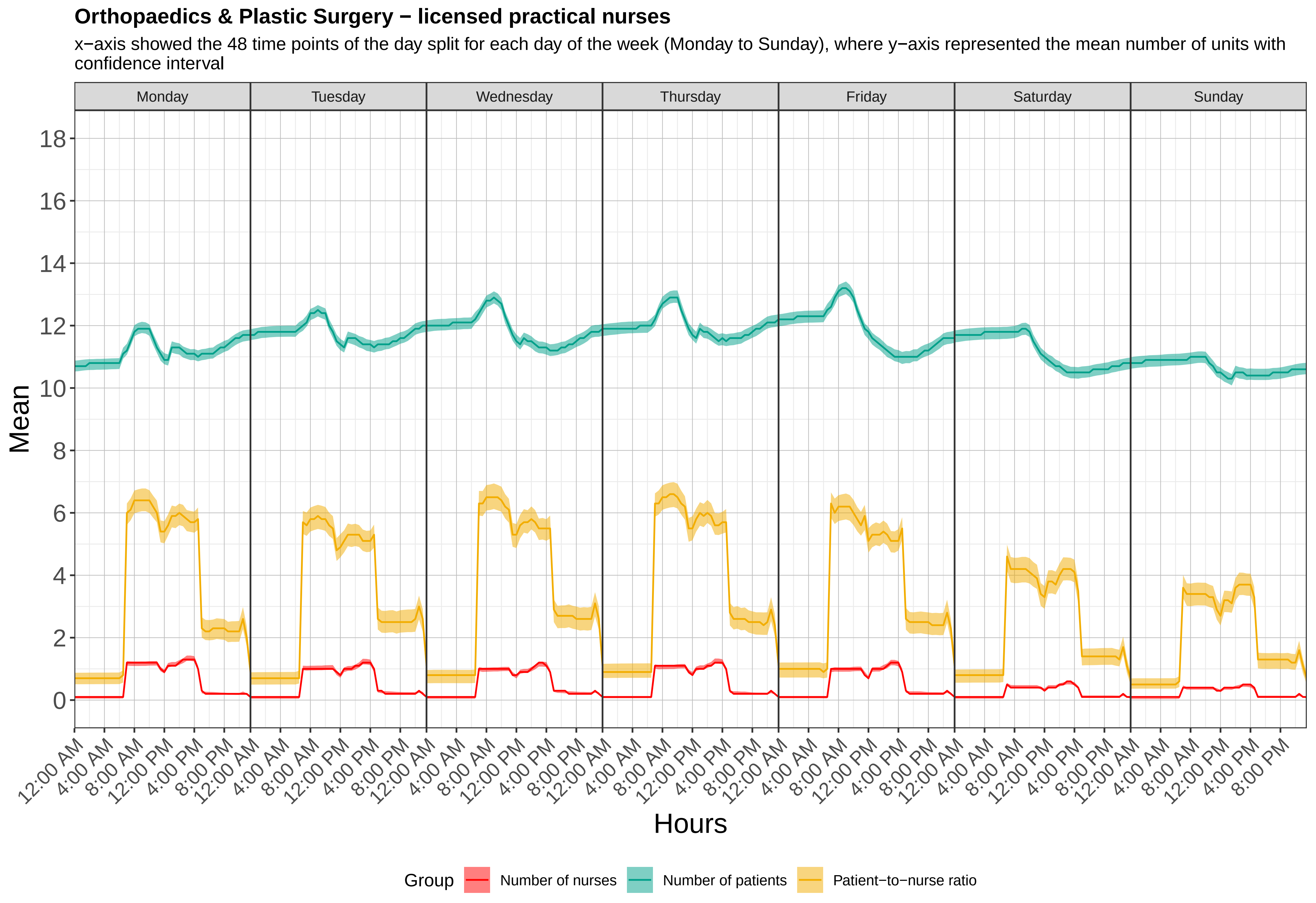
Orthopaedics & Plastic Surgery − licensed practical nurses
x−axis showed the 48 time points of the day split for each day of the week (Monday to Sunday), where y−axis represented the mean number of units with confidence interval
Monday
Tuesday
Wednesday
Thursday
Friday
Saturday
Sunday
Mean
0
2
4
6
8
10
12
14
16
18
12:00 AM
4:00 AM
8:00 AM
12:00 PM
4:00 PM
8:00 PM
Hours
Group
Number of nurses
Number of patients
Patient−to−nurse ratio

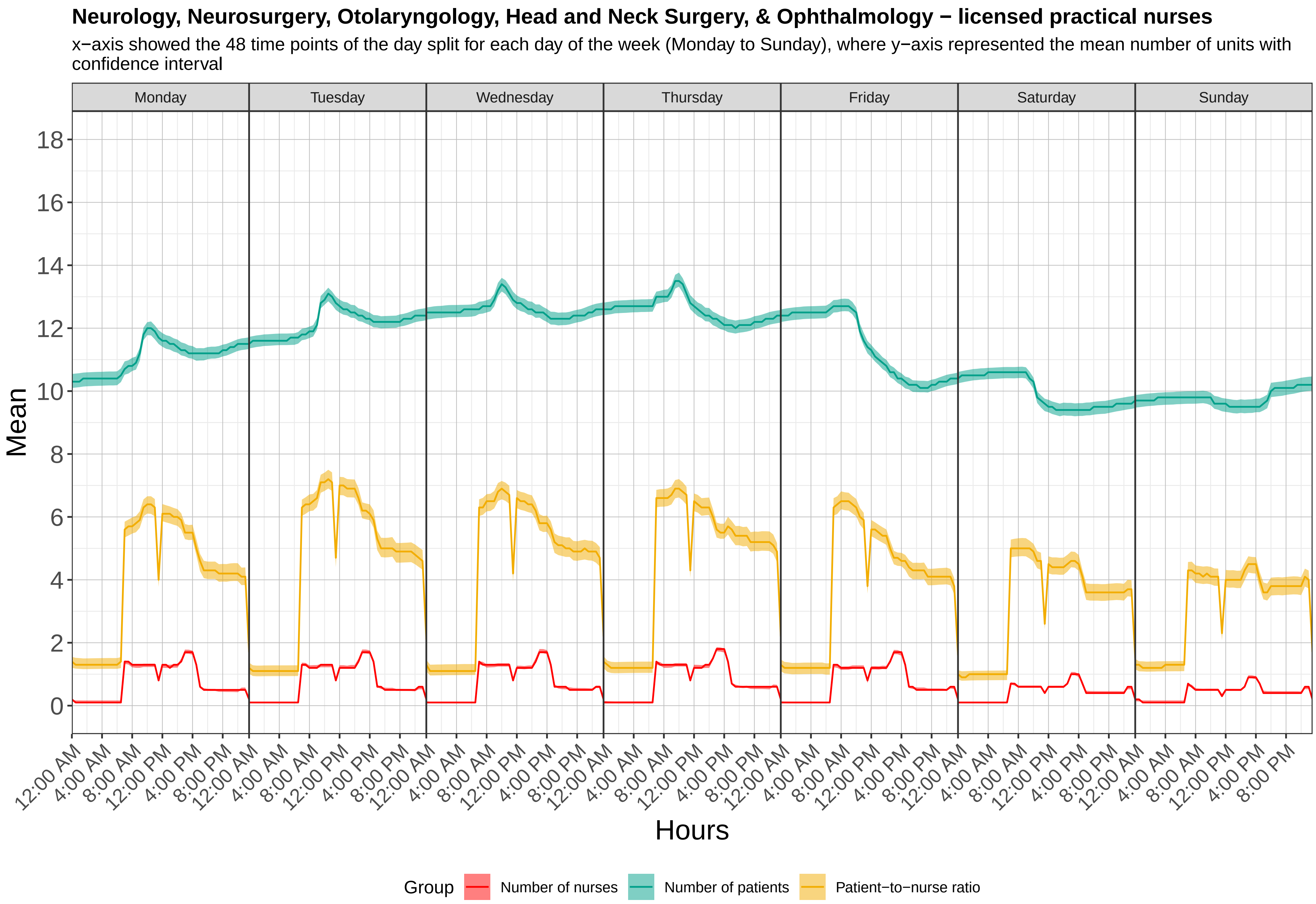

Neurology, Neurosurgery, Otolaryngology, Head and Neck Surgery, & Ophthalmology − licensed practical nurses
x−axis showed the 48 time points of the day split for each day of the week (Monday to Sunday), where y−axis represented the mean number of units with confidence interval
Monday
Tuesday
Wednesday
Thursday
Friday
Saturday
Sunday
Mean
0
2
4
6
8
10
12
14
16
18
12:00 AM
4:00 AM
8:00 AM
12:00 PM
4:00 PM
8:00 PM
Hours
Group
Number of nurses
Number of patients
Patient−to−nurse ratio

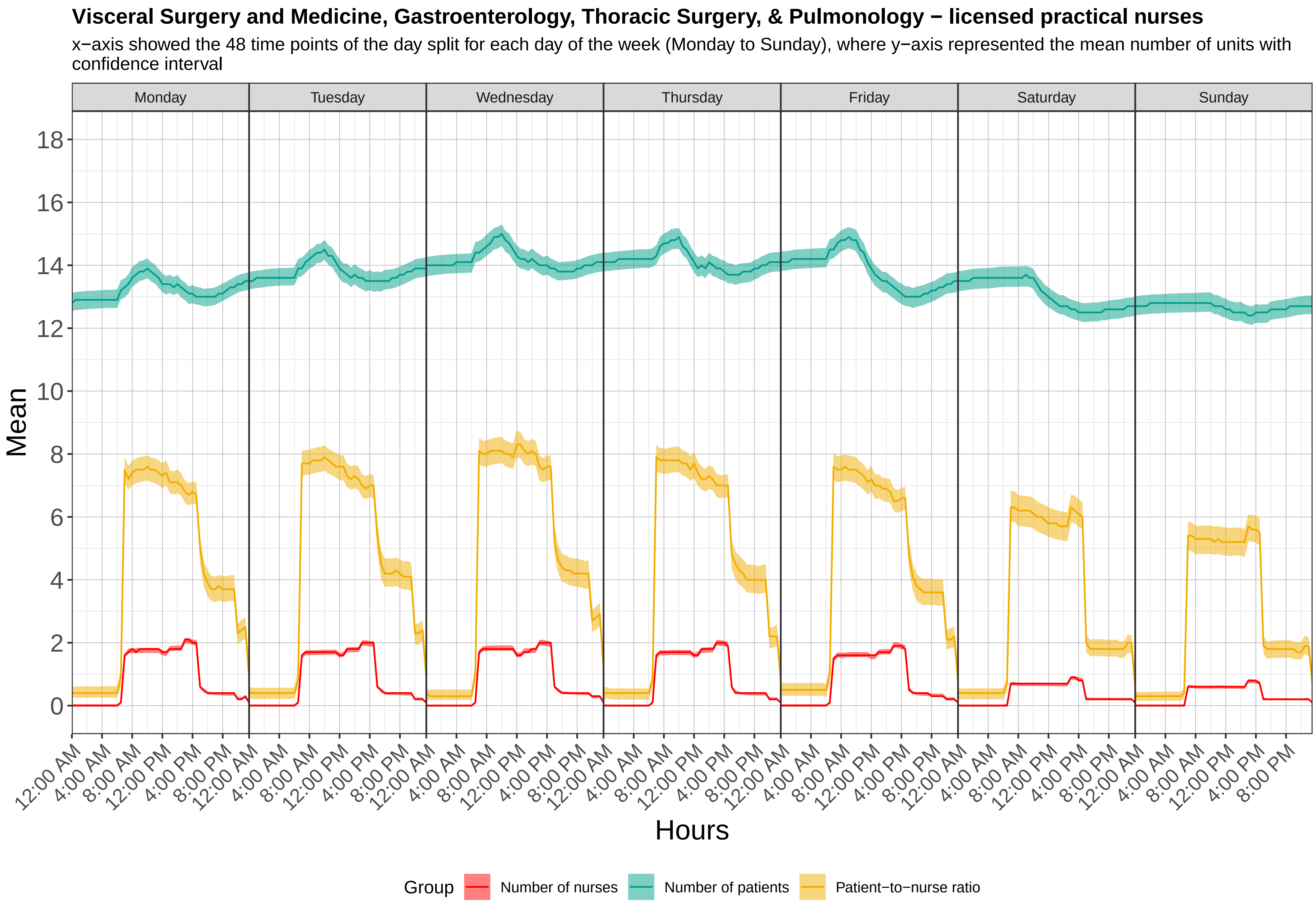

Visceral Surgery and Medicine, Gastroenterology, Thoracic Surgery, & Pulmonology − licensed practical nurses
x−axis showed the 48 time points of the day split for each day of the week (Monday to Sunday), where y−axis represented the mean number of units with confidence interval
Monday
Tuesday
Wednesday
Thursday
Friday
Saturday
Sunday
Mean
0
2
4
6
8
10
12
14
16
18
12:00 AM
4:00 AM
8:00 AM
12:00 PM
4:00 PM
8:00 PM
Hours
Group
Number of nurses
Number of patients
Patient−to−nurse ratio

Dermatology, Urology, Rheumatology, & Nephrology − licensed practical nurses

x−axis showed the 48 time points of the day split for each day of the week (Monday to Sunday), where y−axis represented the mean number of units with confidence interval

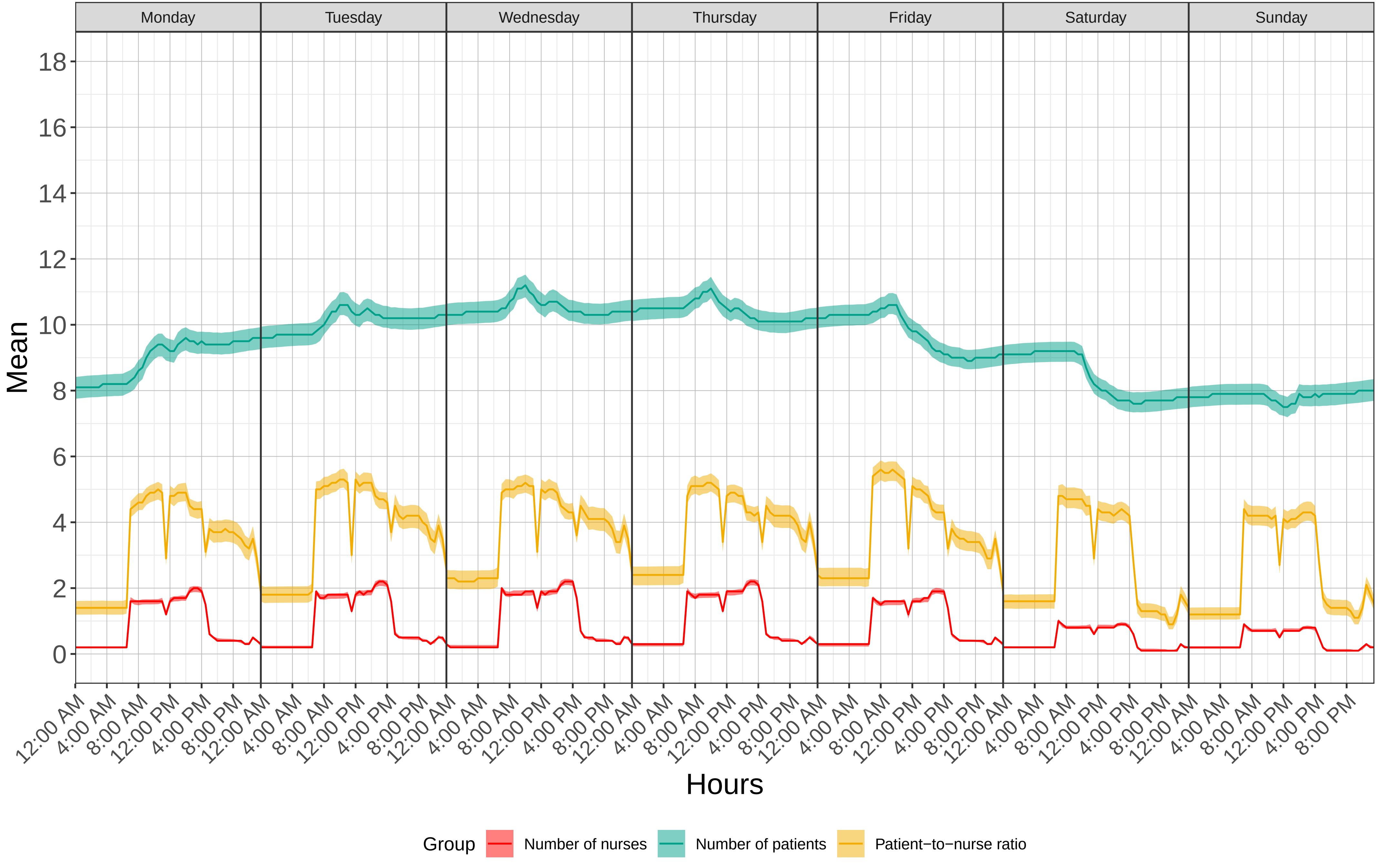

Haematology & Oncology − licensed practical nurses

x−axis showed the 48 time points of the day split for each day of the week (Monday to Sunday), where y−axis represented the mean number of units with confidence interval

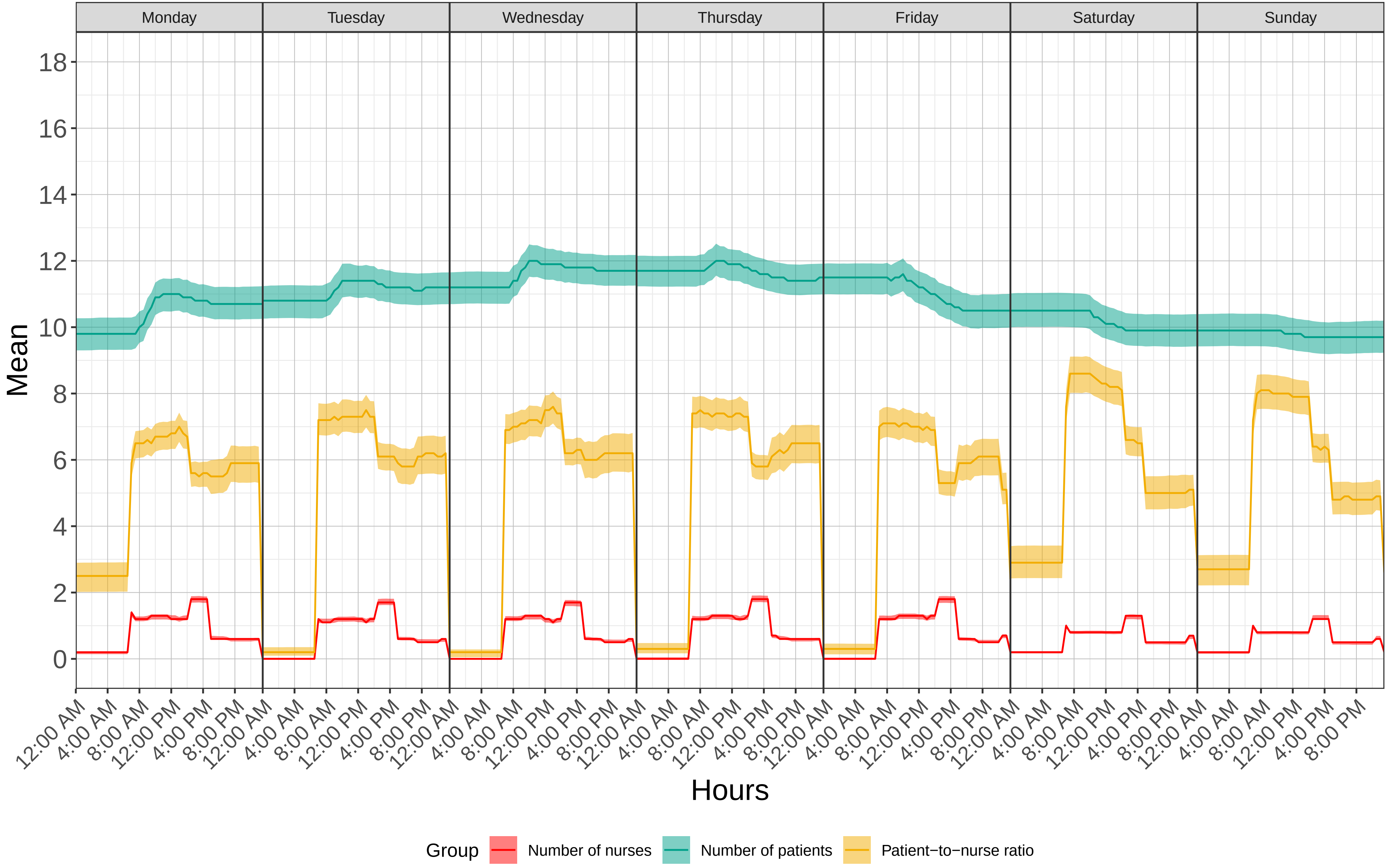

Maternity & Gynecology − licensed practical nurses

x−axis showed the 48 time points of the day split for each day of the week (Monday to Sunday), where y−axis represented the mean number of units with confidence interval

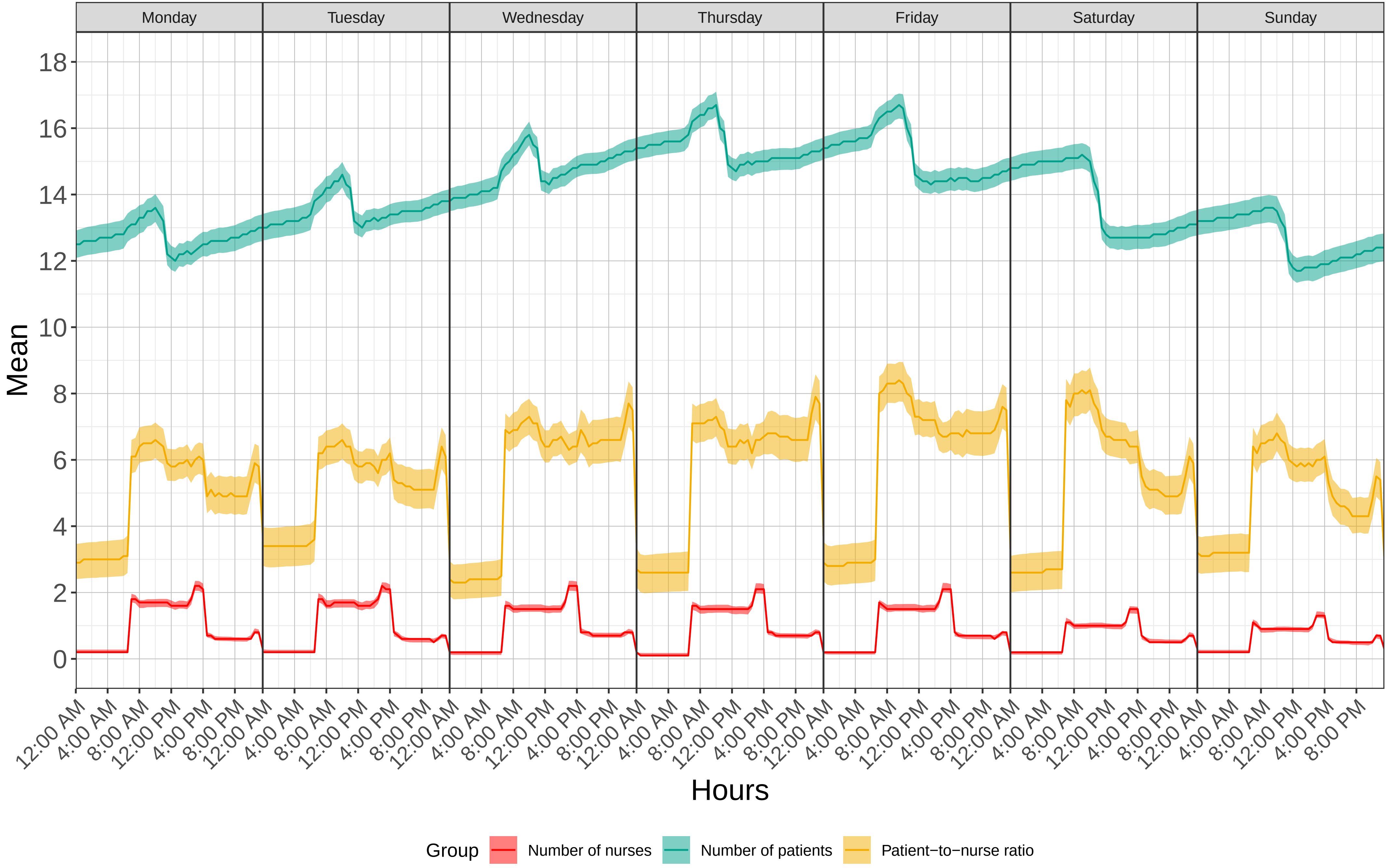

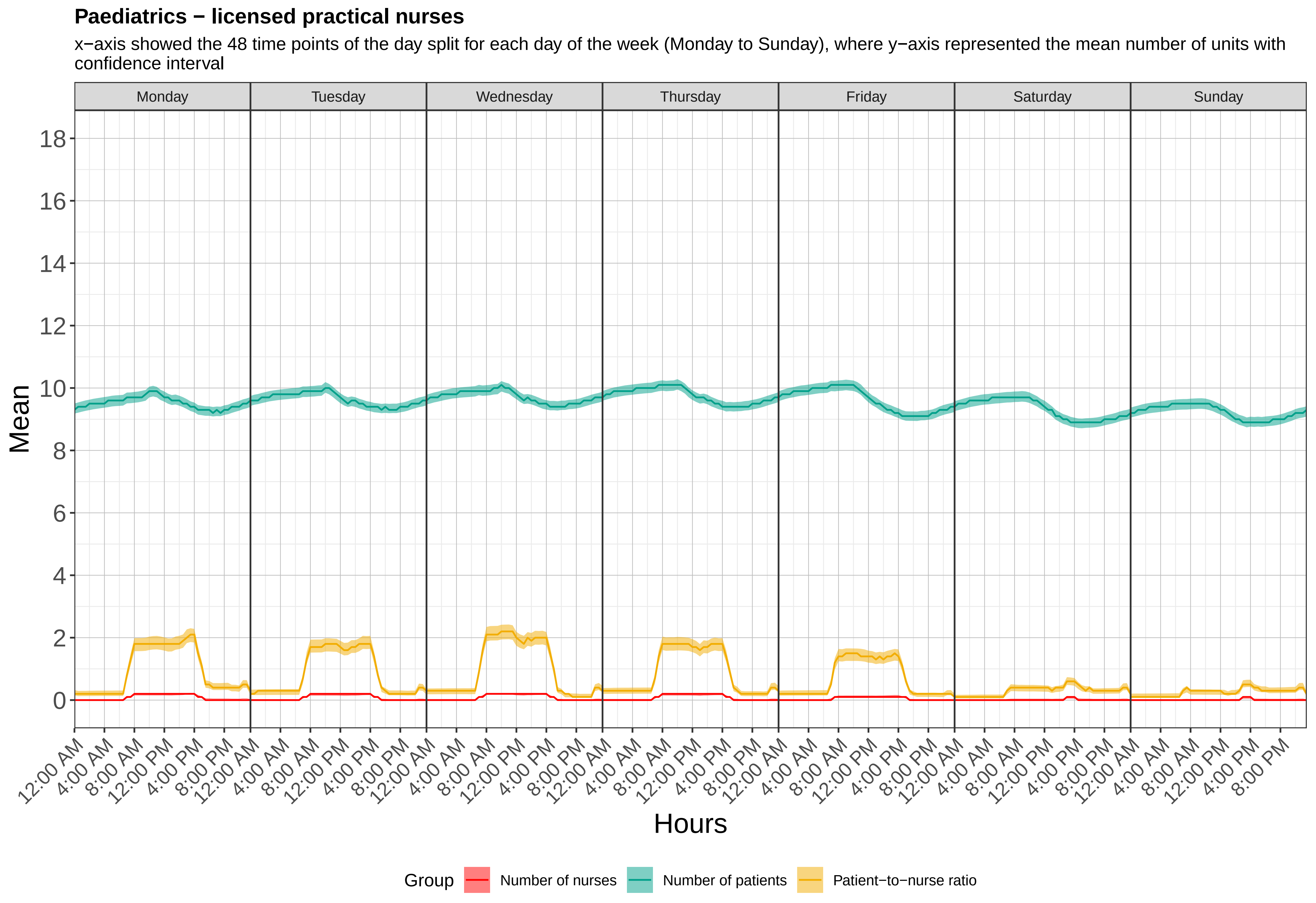

Paediatrics − licensed practical nurses
x−axis showed the 48 time points of the day split for each day of the week (Monday to Sunday), where y−axis represented the mean number of units with confidence interval
Monday
Tuesday
Wednesday
Thursday
Friday
Saturday
Sunday
Mean
0
2
4
6
8
10
12
14
16
18
12:00 AM
4:00 AM
8:00 AM
12:00 PM
4:00 PM
8:00 PM
Hours
Group
Number of nurses
Number of patients
Patient−to−nurse ratio

Intensive Care − licensed practical nurses

x−axis showed the 48 time points of the day split for each day of the week (Monday to Sunday), where y−axis represented the mean number of units with confidence interval

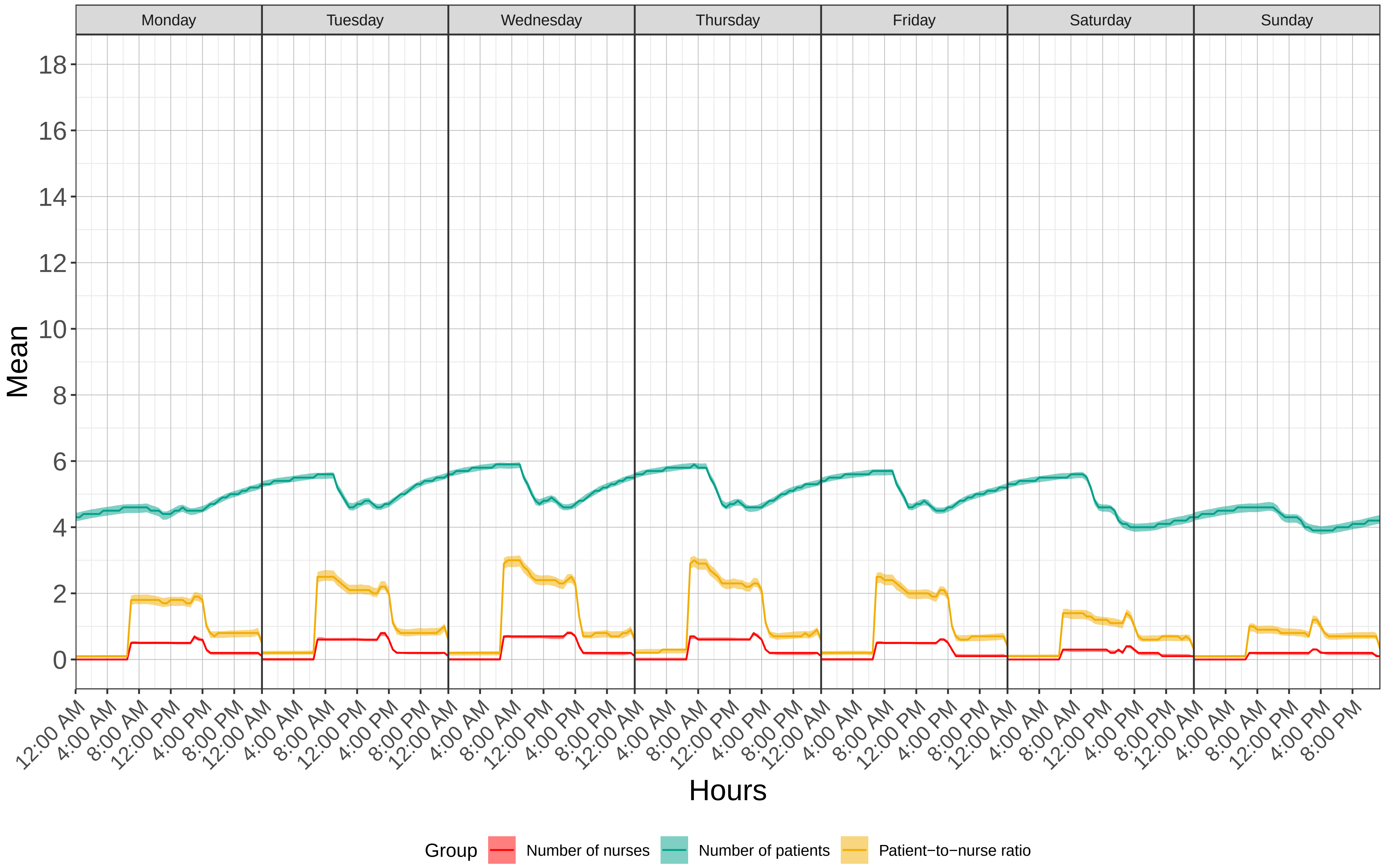

Supplement: Multimedia Appendix 2 [file jmir_v22i4e15554_app2.pdf]
