# Supplementary material for: Longitudinal Study of the Variation in Patient Turnover and Patient-to-Nurse Ratio: Descriptive Analysis of a Swiss University Hospital
Source: J Med Internet Res. 2020 Apr 2;22(4):e15554. doi: 10.2196/15554 (PMC7163415; doi:10.2196/15554)
Supplement: Multimedia Appendix 4 [file jmir_v22i4e15554_app4.pdf]

Internal Medicine

Median of patient−to−nurse ratio for key time points split by weekdays/weekends for each group of nurses together with percentages of shifts with extreme patient−to−nurse ratio

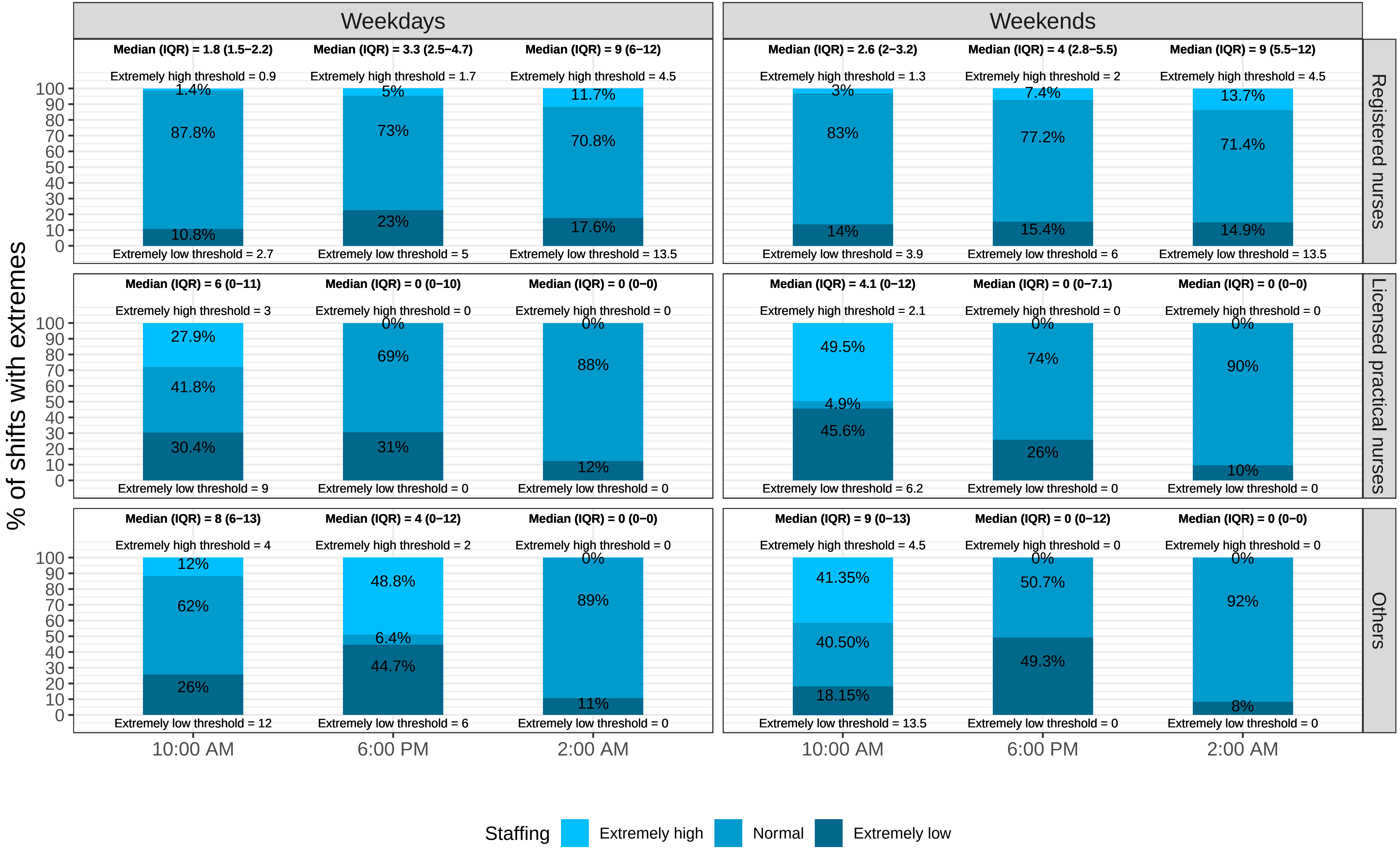

IQR = interquartile range

Cardiology & Cardiovascular Surgery

Median of patient−to−nurse ratio for key time points split by weekdays/weekends for each group of nurses together with percentages of shifts with extreme patient−to−nurse ratio

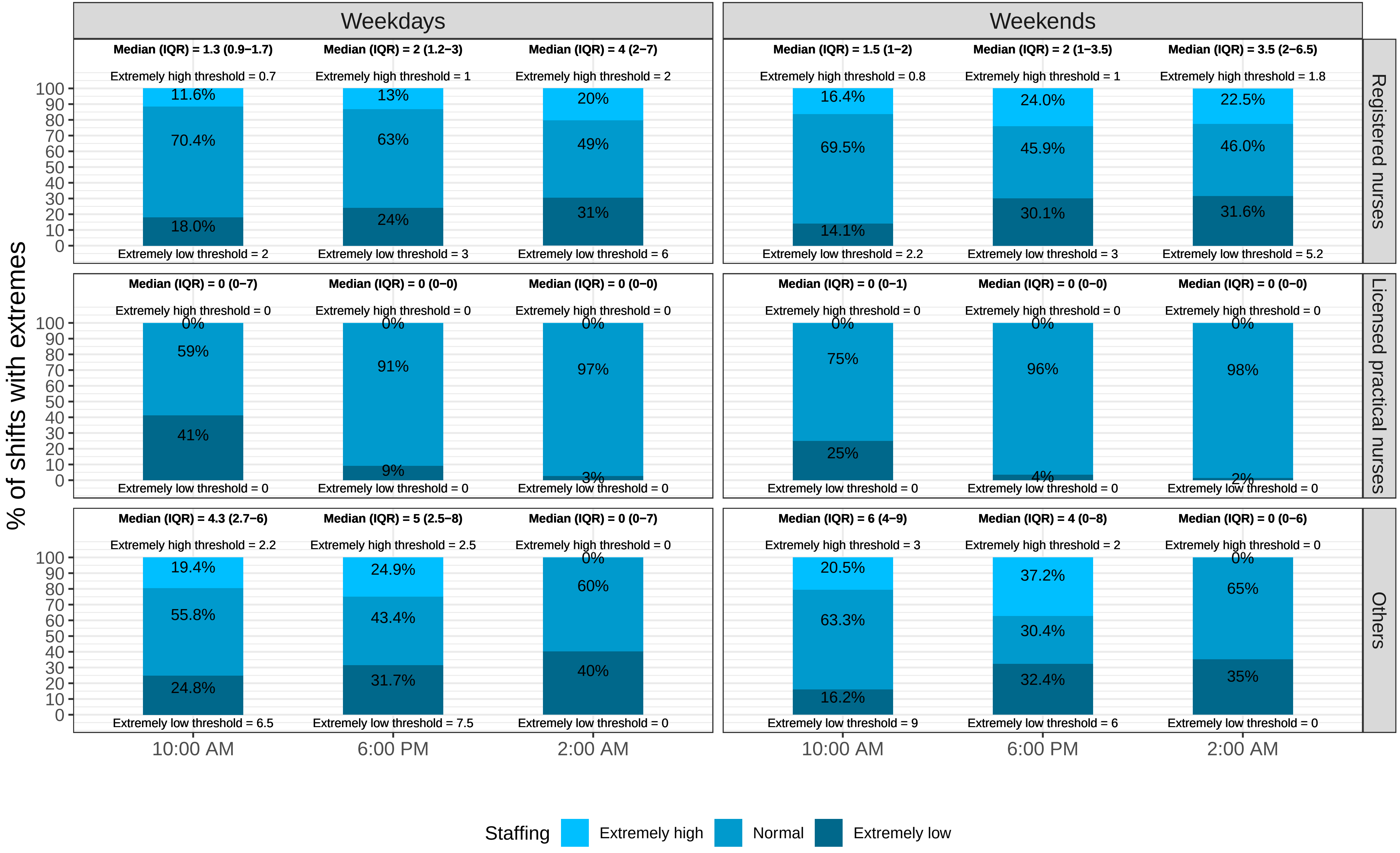

IQR = interquartile range

Orthopaedics & Plastic Surgery

Median of patient−to−nurse ratio for key time points split by weekdays/weekends for each group of nurses together with percentages of shifts with extreme patient−to−nurse ratio

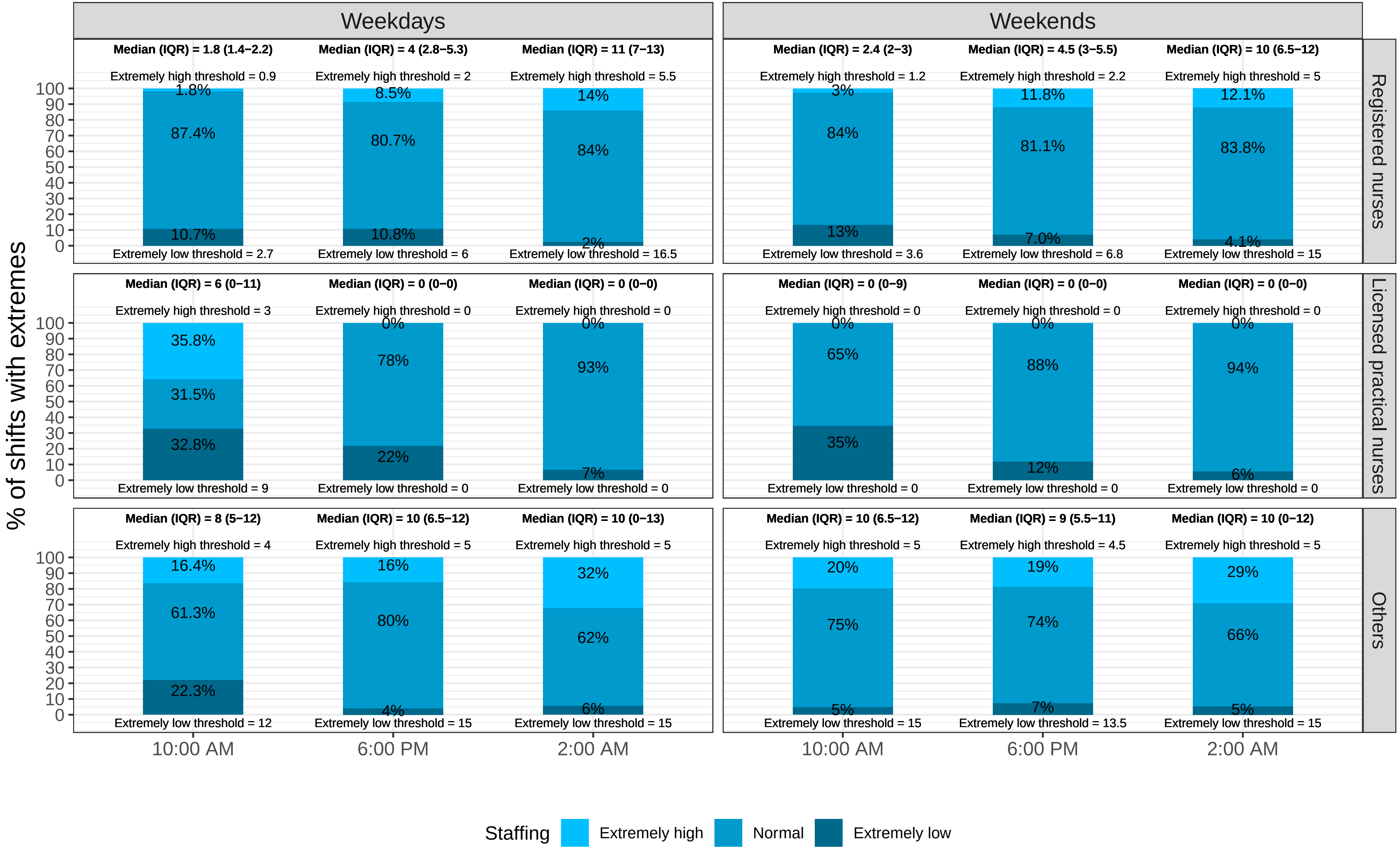

IQR = interquartile range

Neurology, Neurosurgery, Otolaryngology, Head and Neck Surgery, & Ophthalmology

Median of patient−to−nurse ratio for key time points split by weekdays/weekends for each group of nurses together with percentages of shifts with extreme patient−to−nurse ratio

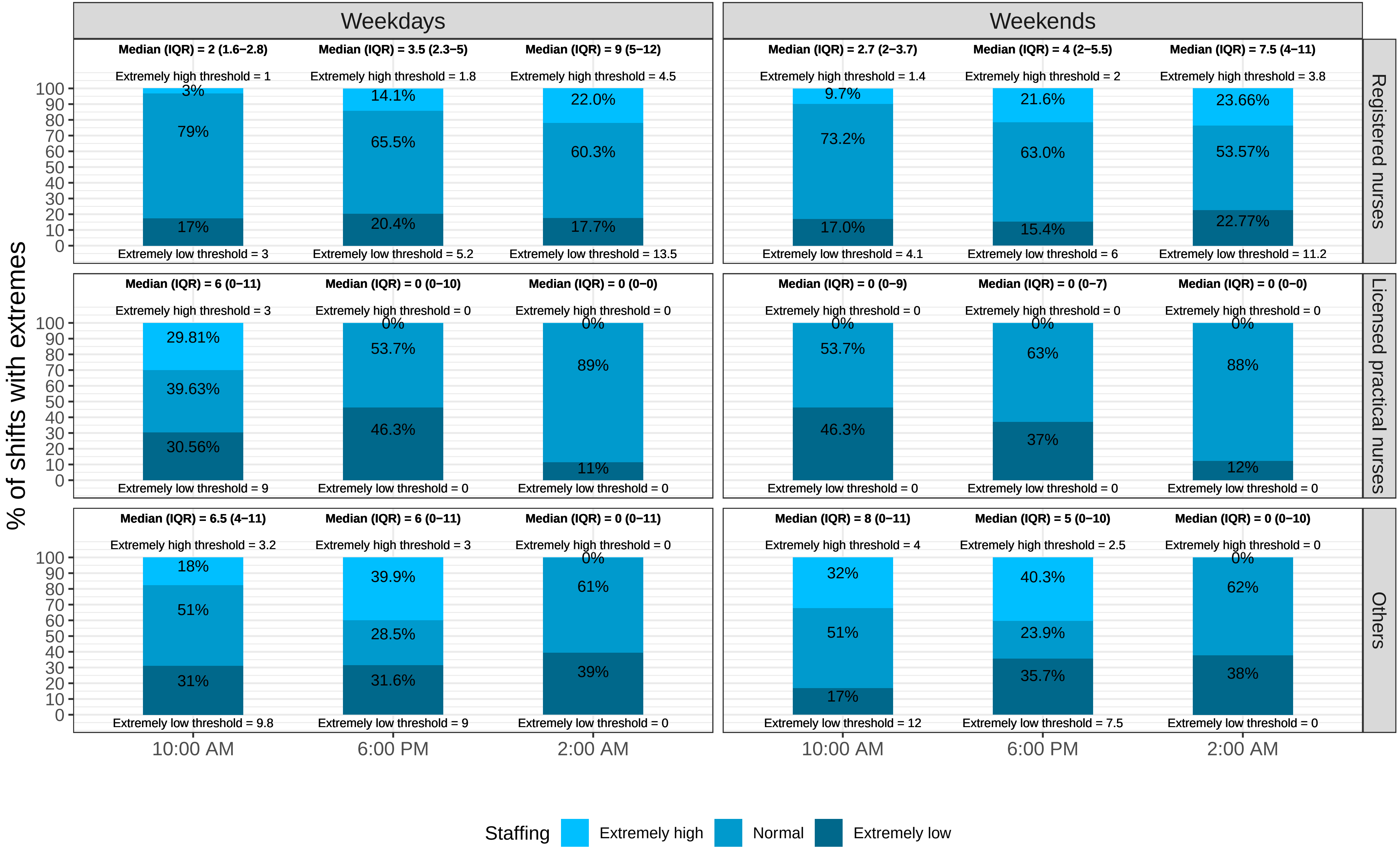

IQR = interquartile range

Visceral Surgery and Medicine, Gastroenterology, Thoracic Surgery, & Pulmonology

Median of patient−to−nurse ratio for key time points split by weekdays/weekends for each group of nurses together with percentages of shifts with extreme patient−to−nurse ratio

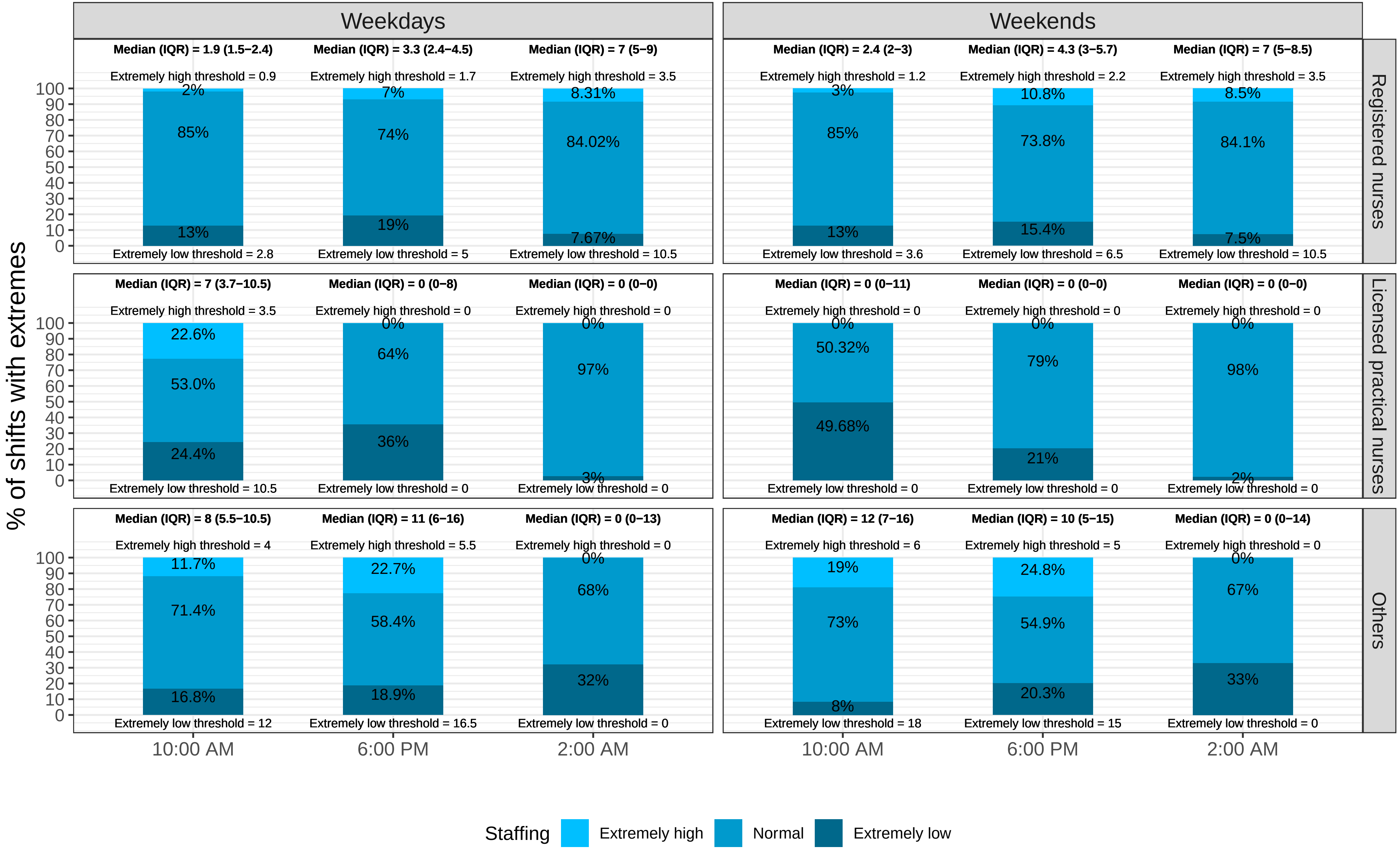

IQR = interquartile range

Dermatology, Urology, Rheumatology, & Nephrology

Median of patient−to−nurse ratio for key time points split by weekdays/weekends for each group of nurses together with percentages of shifts with extreme patient−to−nurse ratio

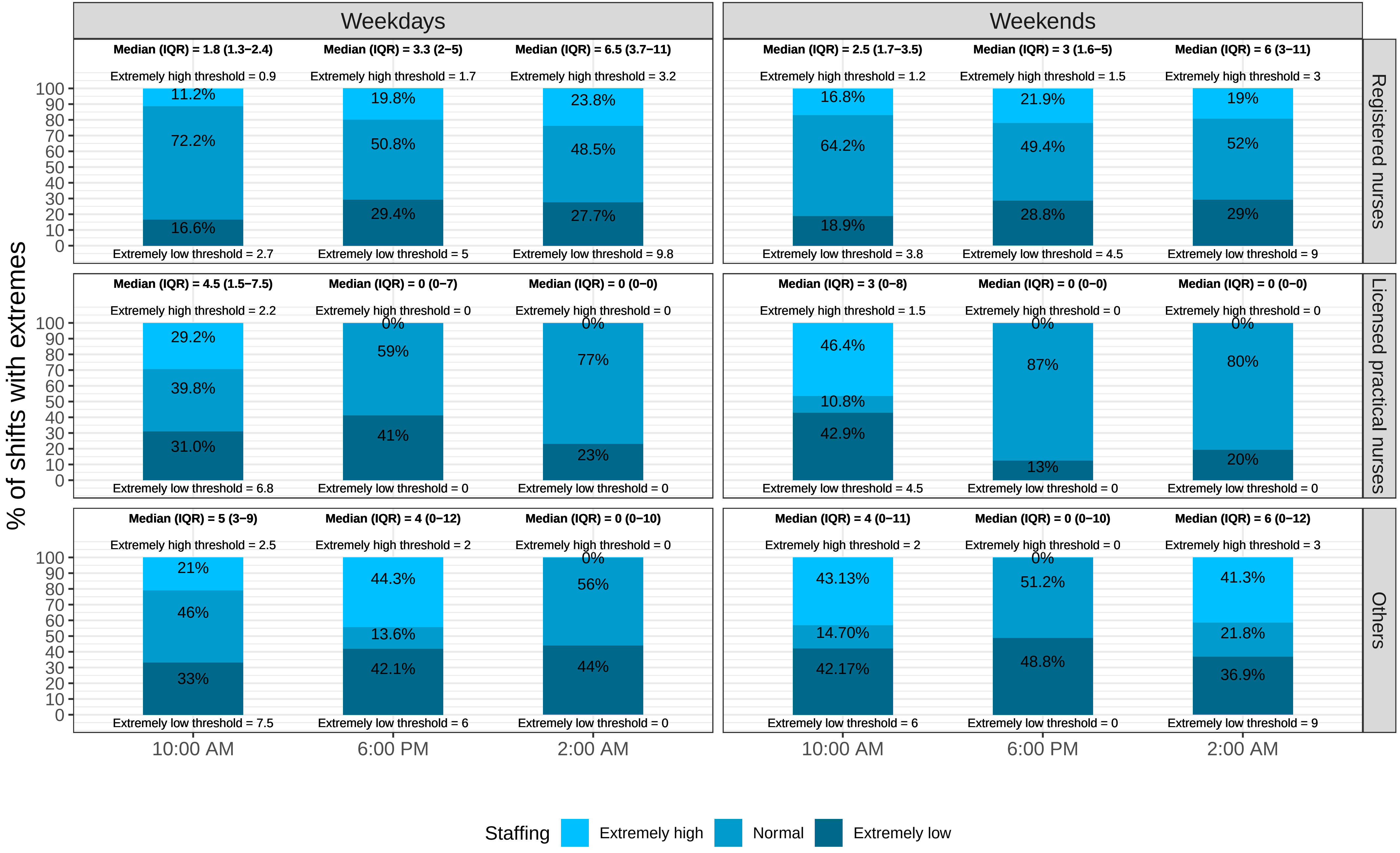

IQR = interquartile range

Haematology & Oncology

Median of patient−to−nurse ratio for key time points split by weekdays/weekends for each group of nurses together with percentages of shifts with extreme patient−to−nurse ratio

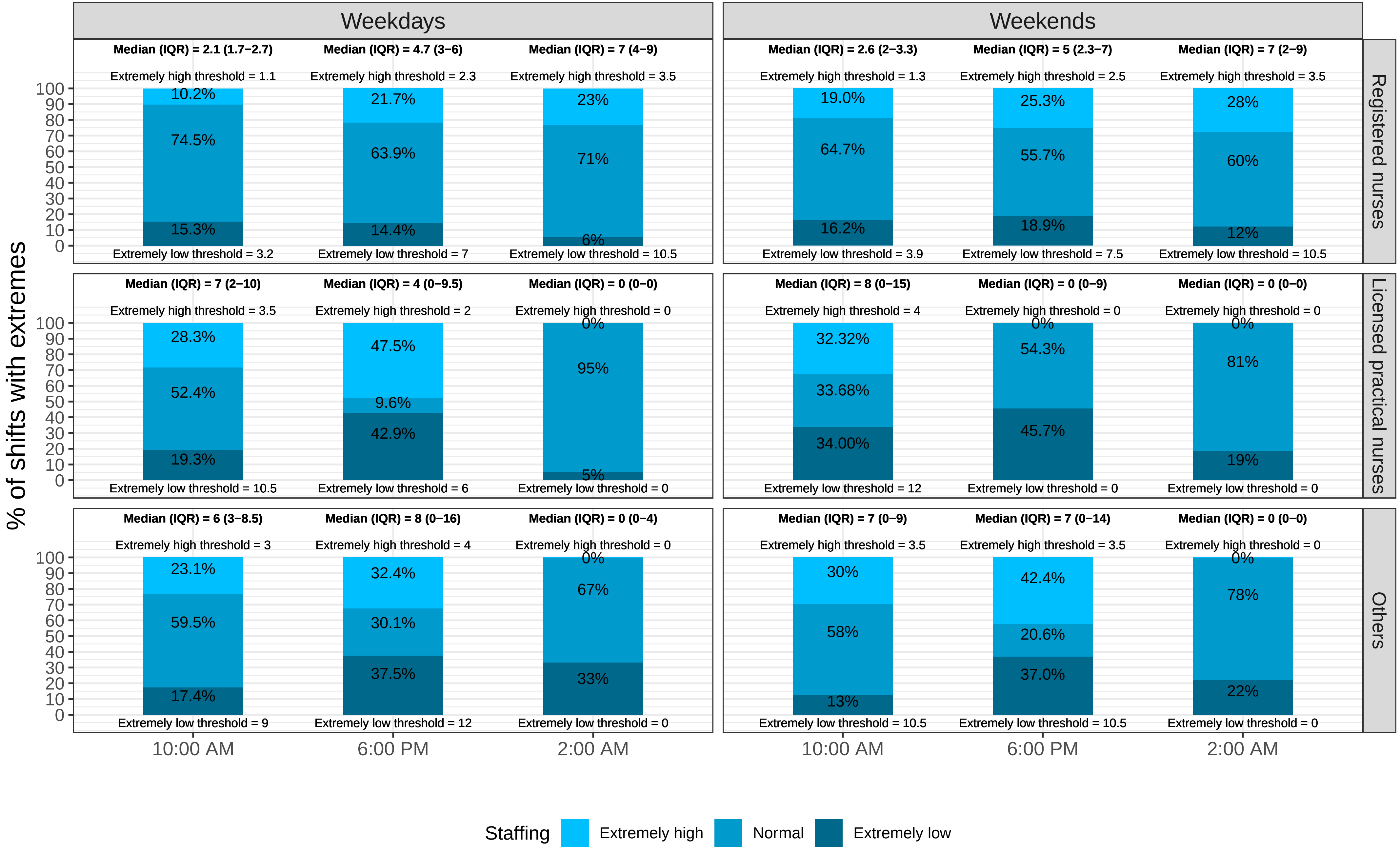

IQR = interquartile range

Maternity & Gynecology

Median of patient−to−nurse ratio for key time points split by weekdays/weekends for each group of nurses together with percentages of shifts with extreme patient−to−nurse ratio

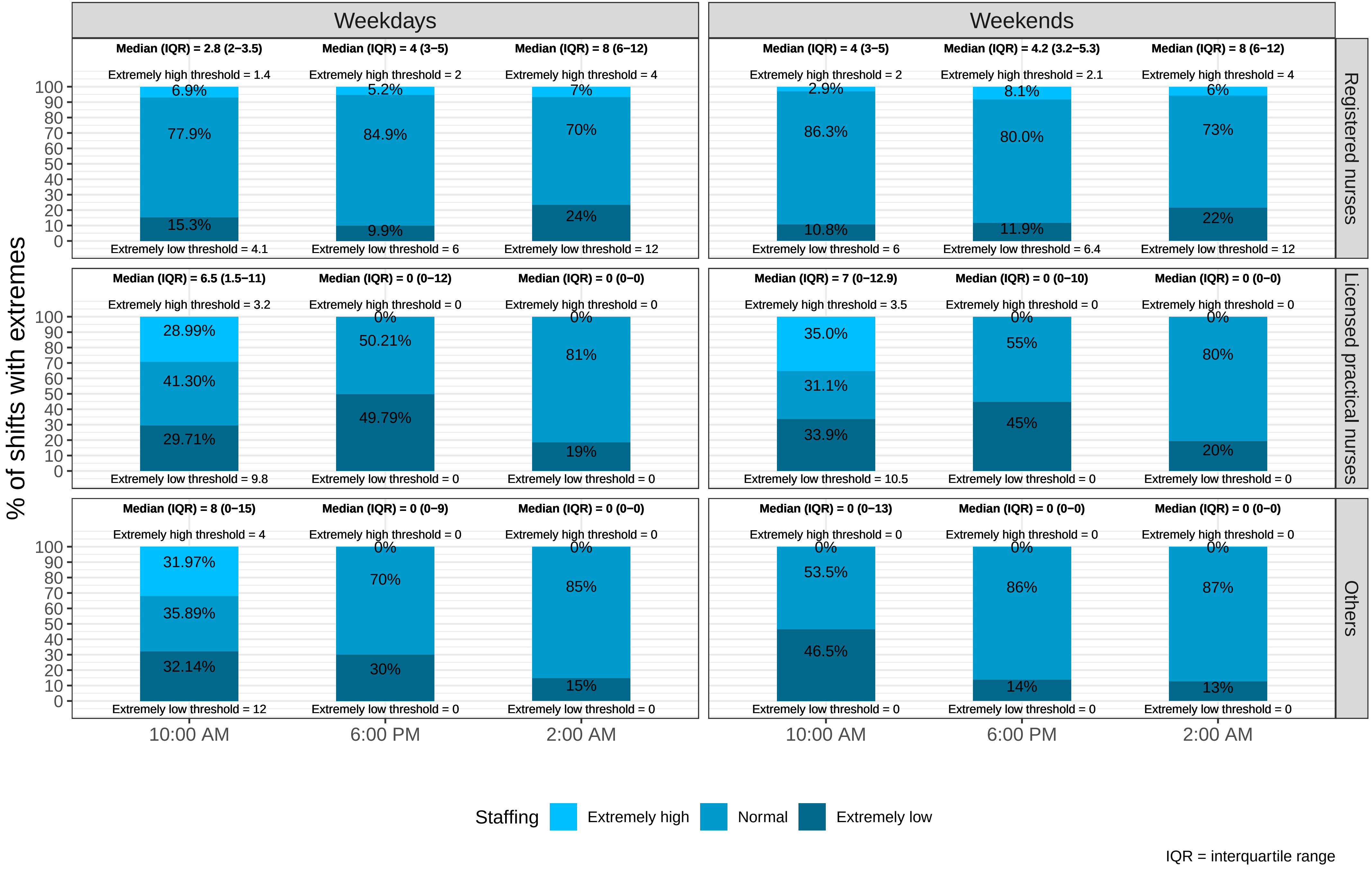

Paediatrics

Median of patient−to−nurse ratio for key time points split by weekdays/weekends for each group of nurses together with percentages of shifts with extreme patient−to−nurse ratio

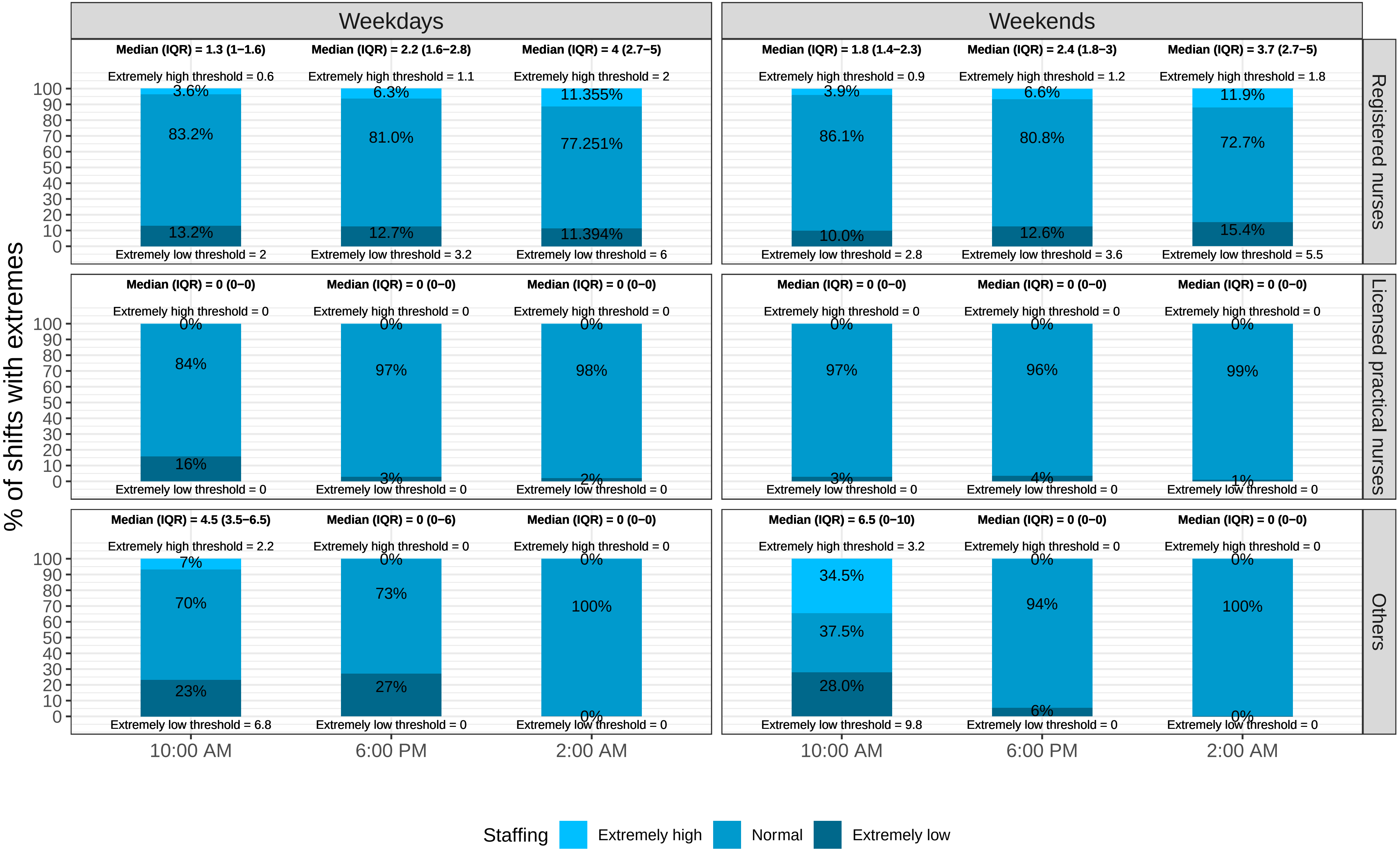

IQR = interquartile range

Intensive Care

Median of patient−to−nurse ratio for key time points split by weekdays/weekends for each group of nurses together with percentages of shifts with extreme patient−to−nurse ratio

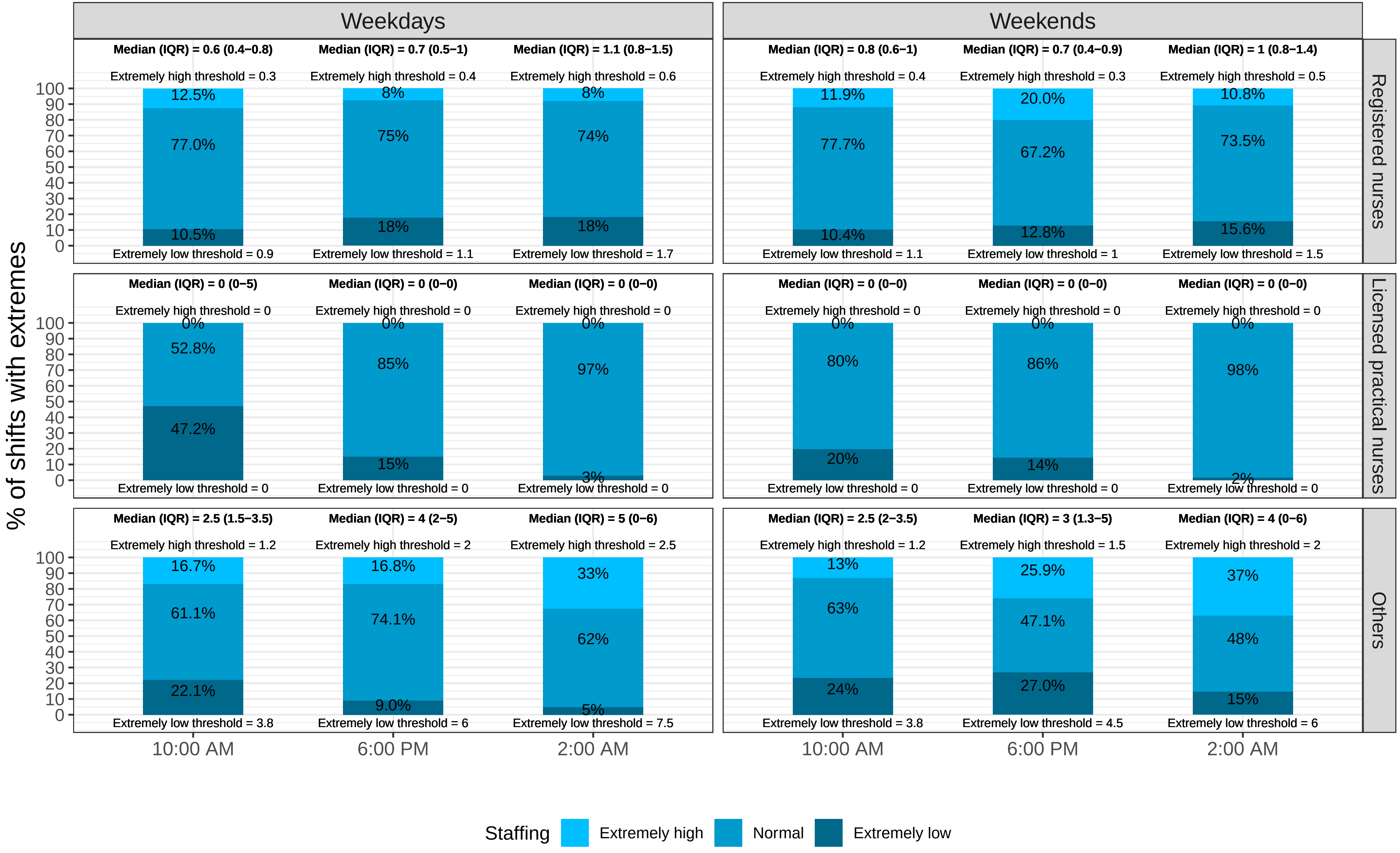

IQR = interquartile range
