# Supplementary material for: Longitudinal Study of the Variation in Patient Turnover and Patient-to-Nurse Ratio: Descriptive Analysis of a Swiss University Hospital
Source: J Med Internet Res. 2020 Apr 2;22(4):e15554. doi: 10.2196/15554 (PMC7163415; doi:10.2196/15554)

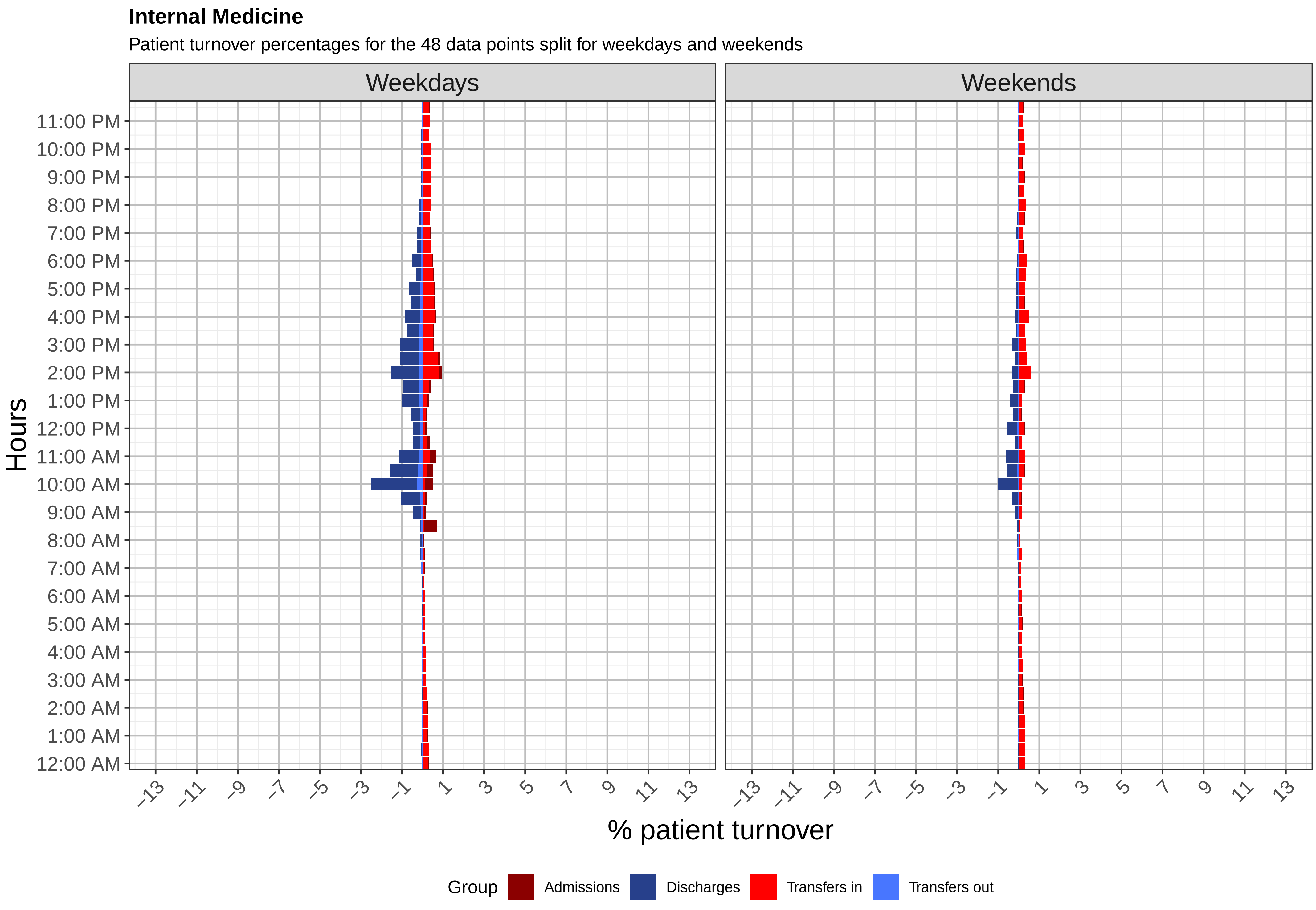
Internal Medicine
Patient turnover percentages for the 48 data points split for weekdays and weekends
Weekdays
Weekends
Hours
11:00 PM
10:00 PM
9:00 PM
8:00 PM
7:00 PM
6:00 PM
5:00 PM
4:00 PM
3:00 PM
2:00 PM
1:00 PM
12:00 PM
11:00 AM
10:00 AM
9:00 AM
8:00 AM
7:00 AM
6:00 AM
5:00 AM
4:00 AM
3:00 AM
2:00 AM
1:00 AM
12:00 AM
−13 −11 −9 −7 −5 −3 −1 1 3 5 7 9 11 13
% patient turnover
Group
Admissions
Discharges
Transfers in
Transfers out

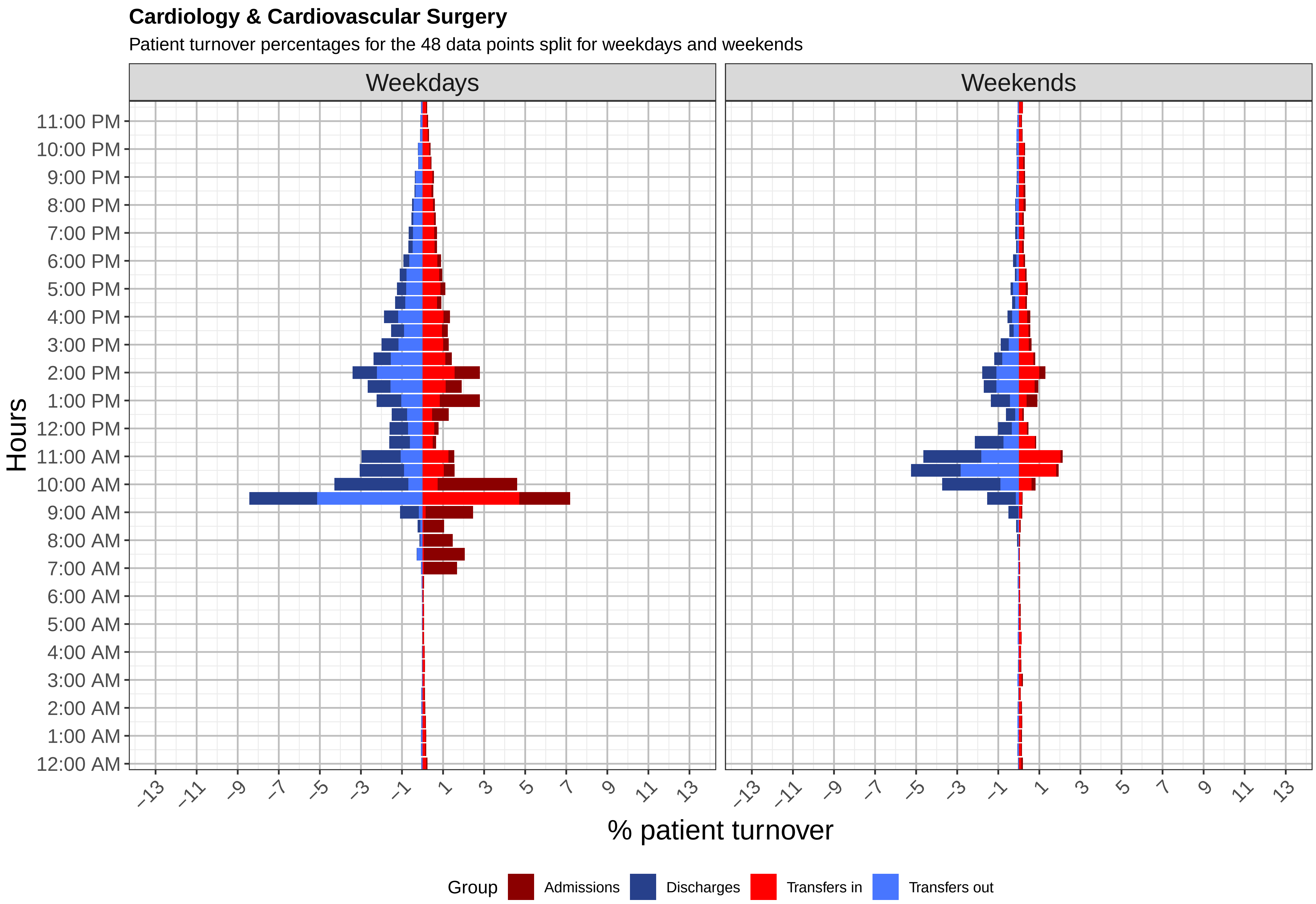

Cardiology & Cardiovascular Surgery
Patient turnover percentages for the 48 data points split for weekdays and weekends
Weekdays
Weekends
Hours
11:00 PM
10:00 PM
9:00 PM
8:00 PM
7:00 PM
6:00 PM
5:00 PM
4:00 PM
3:00 PM
2:00 PM
1:00 PM
12:00 PM
11:00 AM
10:00 AM
9:00 AM
8:00 AM
7:00 AM
6:00 AM
5:00 AM
4:00 AM
3:00 AM
2:00 AM
1:00 AM
12:00 AM
–13
–11
–9
–7
–5
–3
–1
1
3
5
7
9
11
13
% patient turnover
Group
Admissions
Discharges
Transfers in
Transfers out

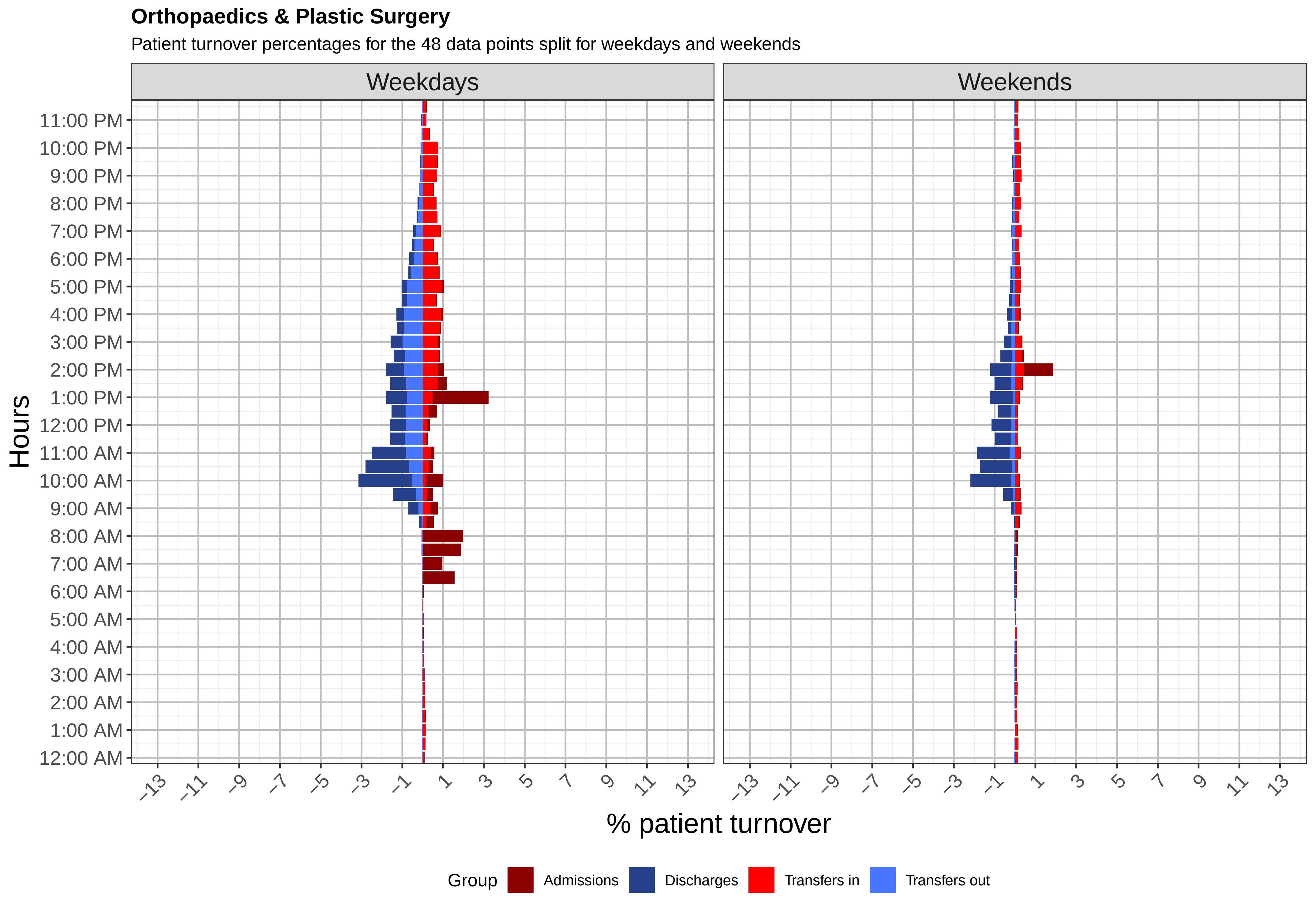

Orthopaedics & Plastic Surgery
Patient turnover percentages for the 48 data points split for weekdays and weekends
Weekdays
Weekends
Hours
11:00 PM
10:00 PM
9:00 PM
8:00 PM
7:00 PM
6:00 PM
5:00 PM
4:00 PM
3:00 PM
2:00 PM
1:00 PM
12:00 PM
11:00 AM
10:00 AM
9:00 AM
8:00 AM
7:00 AM
6:00 AM
5:00 AM
4:00 AM
3:00 AM
2:00 AM
1:00 AM
12:00 AM
−13
−11
−9
−7
−5
−3
−1
1
3
5
7
9
11
13
% patient turnover
Group
Admissions
Discharges
Transfers in
Transfers out

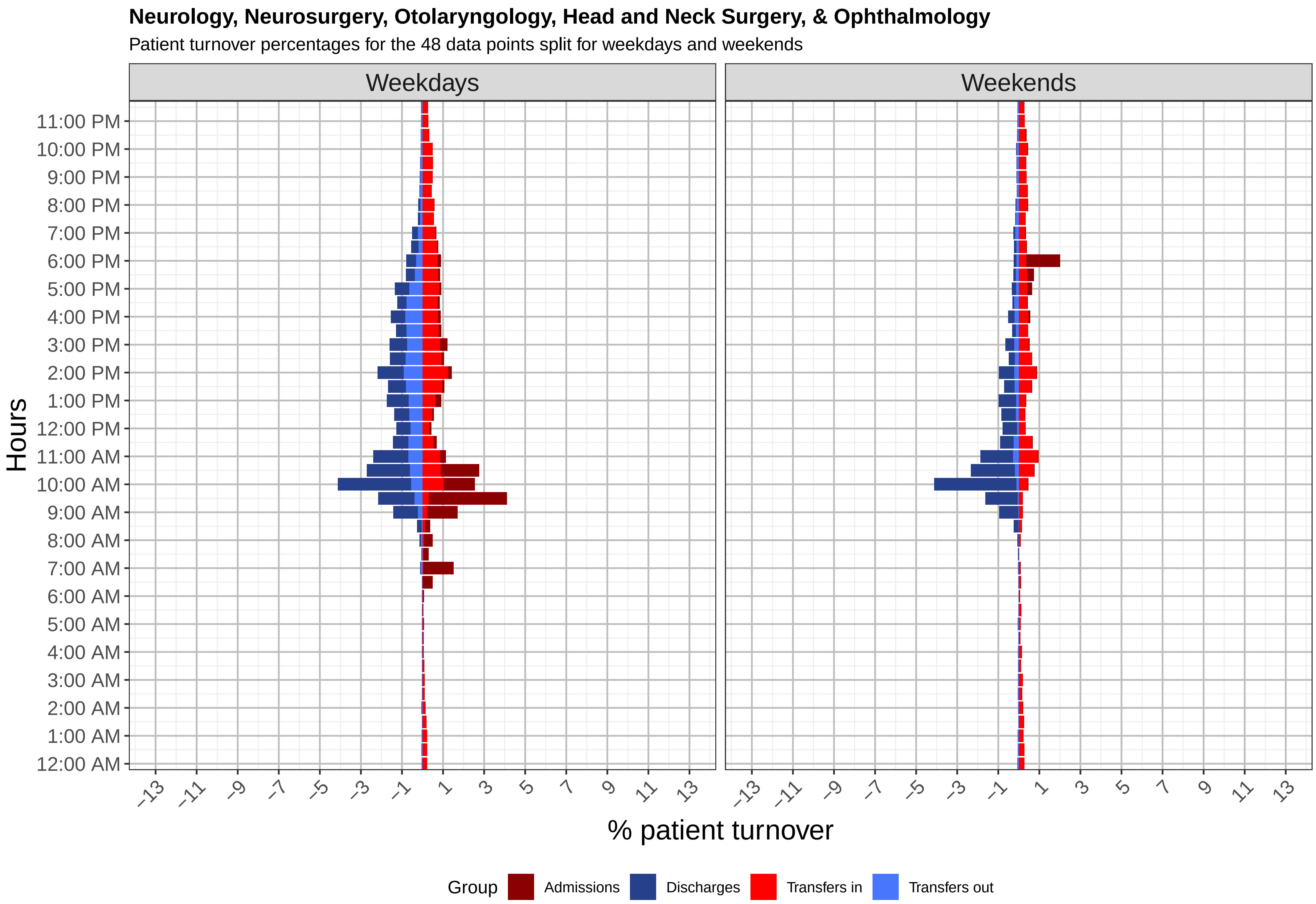

Neurology, Neurosurgery, Otolaryngology, Head and Neck Surgery, & Ophthalmology
Patient turnover percentages for the 48 data points split for weekdays and weekends
Weekdays
Weekends
Hours
11:00 PM
10:00 PM
9:00 PM
8:00 PM
7:00 PM
6:00 PM
5:00 PM
4:00 PM
3:00 PM
2:00 PM
1:00 PM
12:00 PM
11:00 AM
10:00 AM
9:00 AM
8:00 AM
7:00 AM
6:00 AM
5:00 AM
4:00 AM
3:00 AM
2:00 AM
1:00 AM
12:00 AM
−13 −11 −9 −7 −5 −3 −1 1 3 5 7 9 11 13
% patient turnover
Group
Admissions
Discharges
Transfers in
Transfers out

Visceral Surgery and Medicine, Gastroenterology, Thoracic Surgery, & Pulmonology

Patient turnover percentages for the 48 data points split for weekdays and weekends

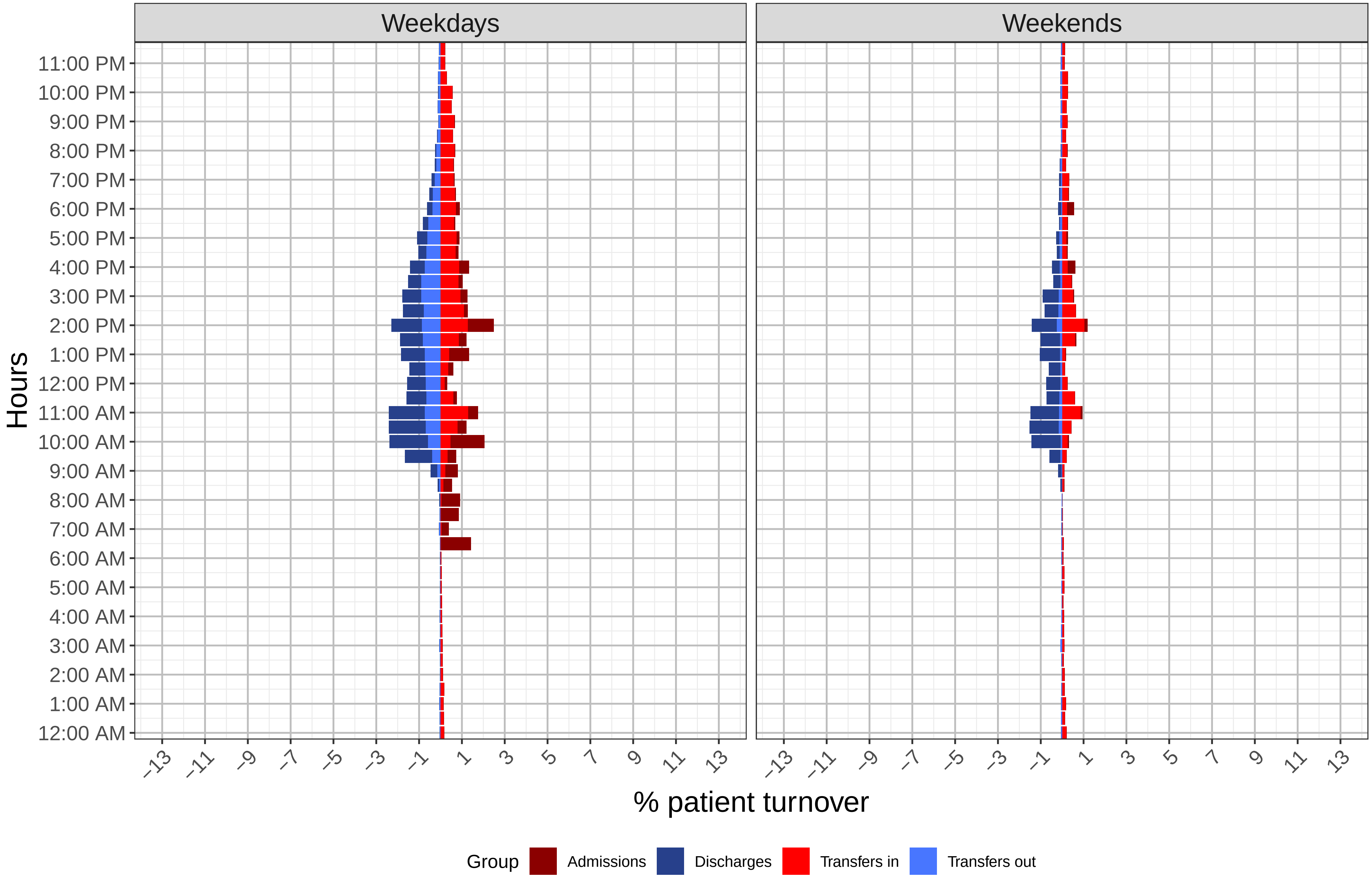

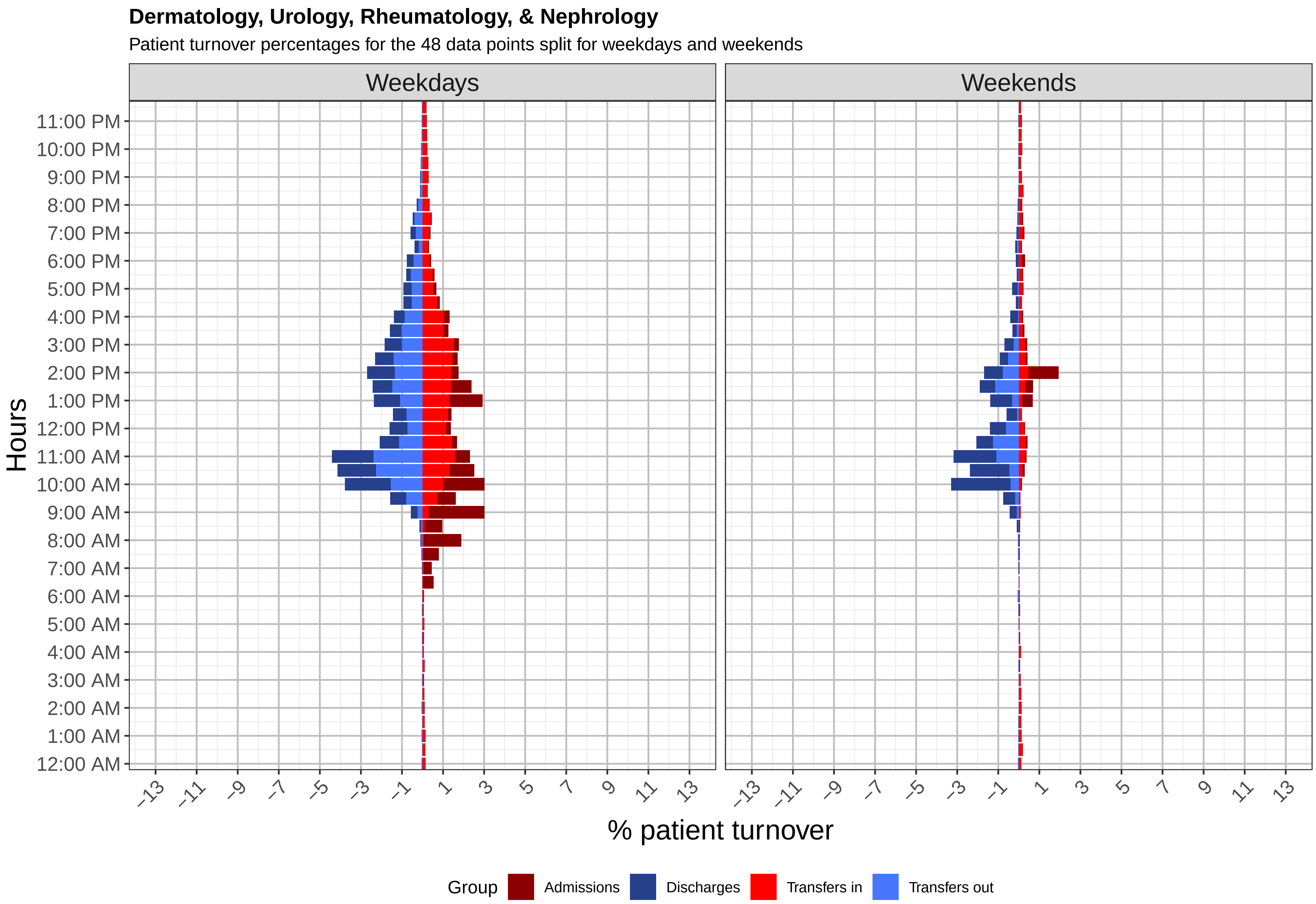

Dermatology, Urology, Rheumatology, & Nephrology
Patient turnover percentages for the 48 data points split for weekdays and weekends
Weekdays
Weekends
Hours
11:00 PM
10:00 PM
9:00 PM
8:00 PM
7:00 PM
6:00 PM
5:00 PM
4:00 PM
3:00 PM
2:00 PM
1:00 PM
12:00 PM
11:00 AM
10:00 AM
9:00 AM
8:00 AM
7:00 AM
6:00 AM
5:00 AM
4:00 AM
3:00 AM
2:00 AM
1:00 AM
12:00 AM
−13 −11 −9 −7 −5 −3 −1 1 3 5 7 9 11 13
% patient turnover
Group
Admissions
Discharges
Transfers in
Transfers out

Haematology & Oncology

Patient turnover percentages for the 48 data points split for weekdays and weekends

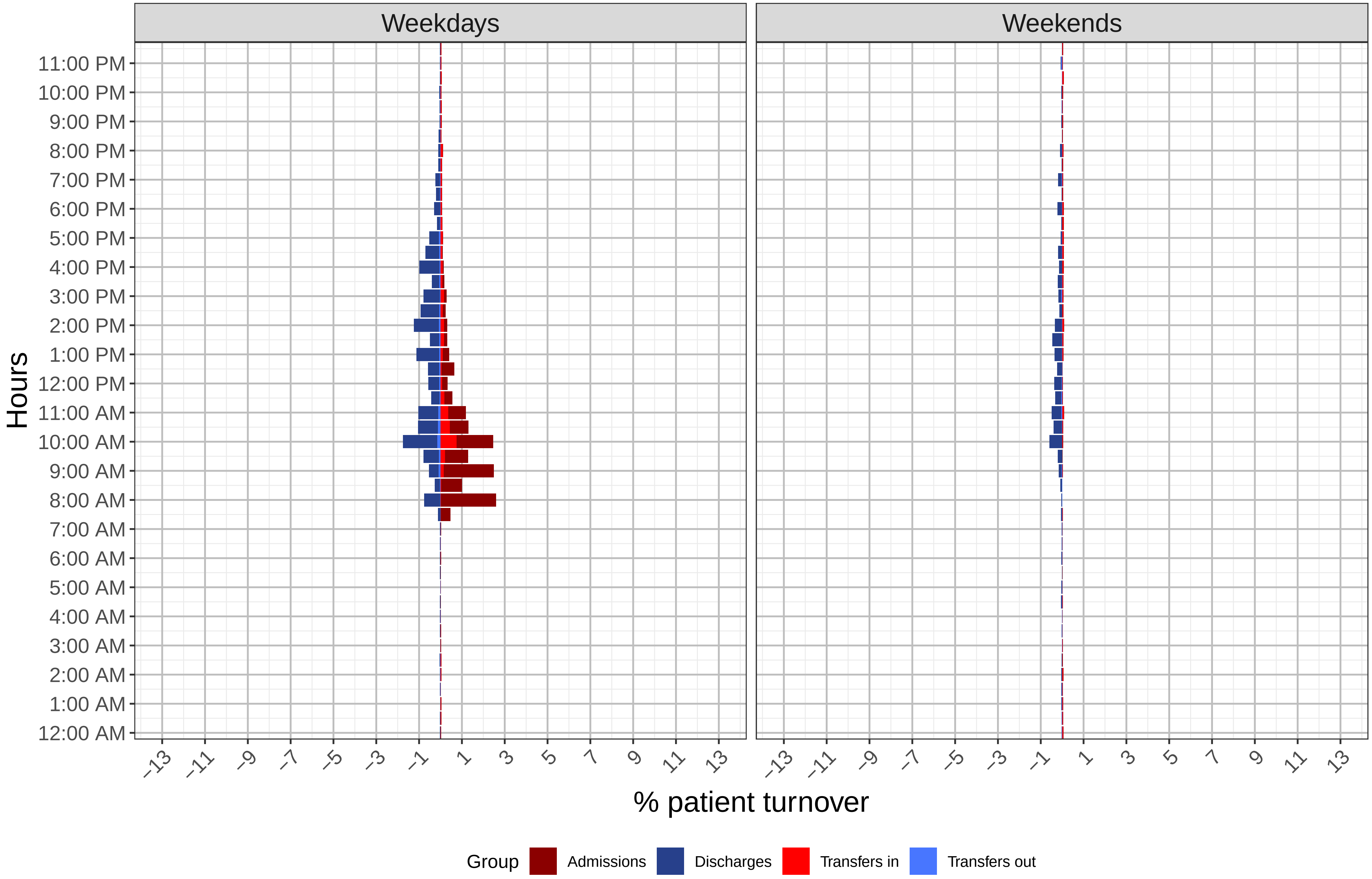

Maternity & Gynecology

Patient turnover percentages for the 48 data points split for weekdays and weekends

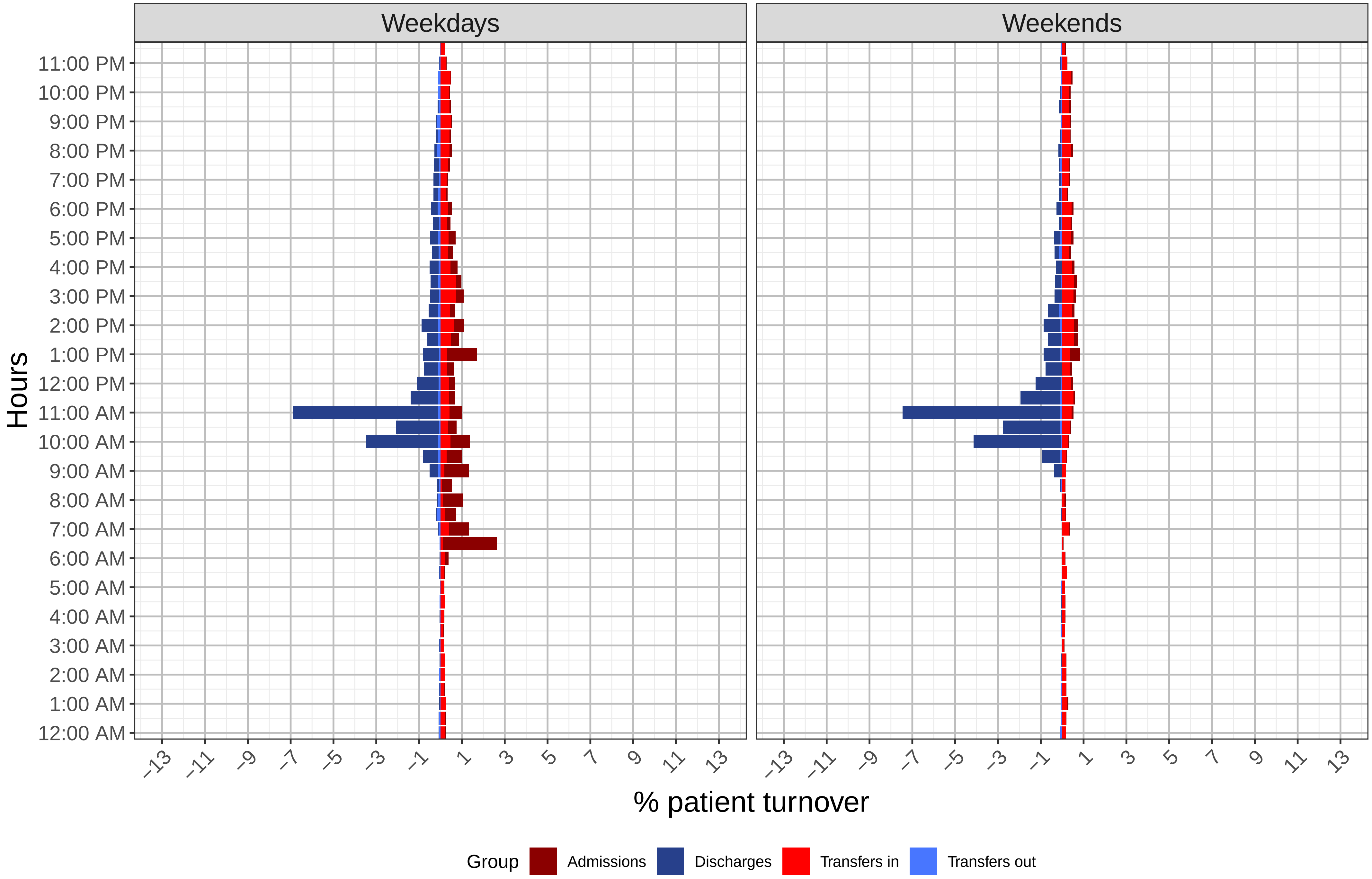

Paediatrics

Patient turnover percentages for the 48 data points split for weekdays and weekends

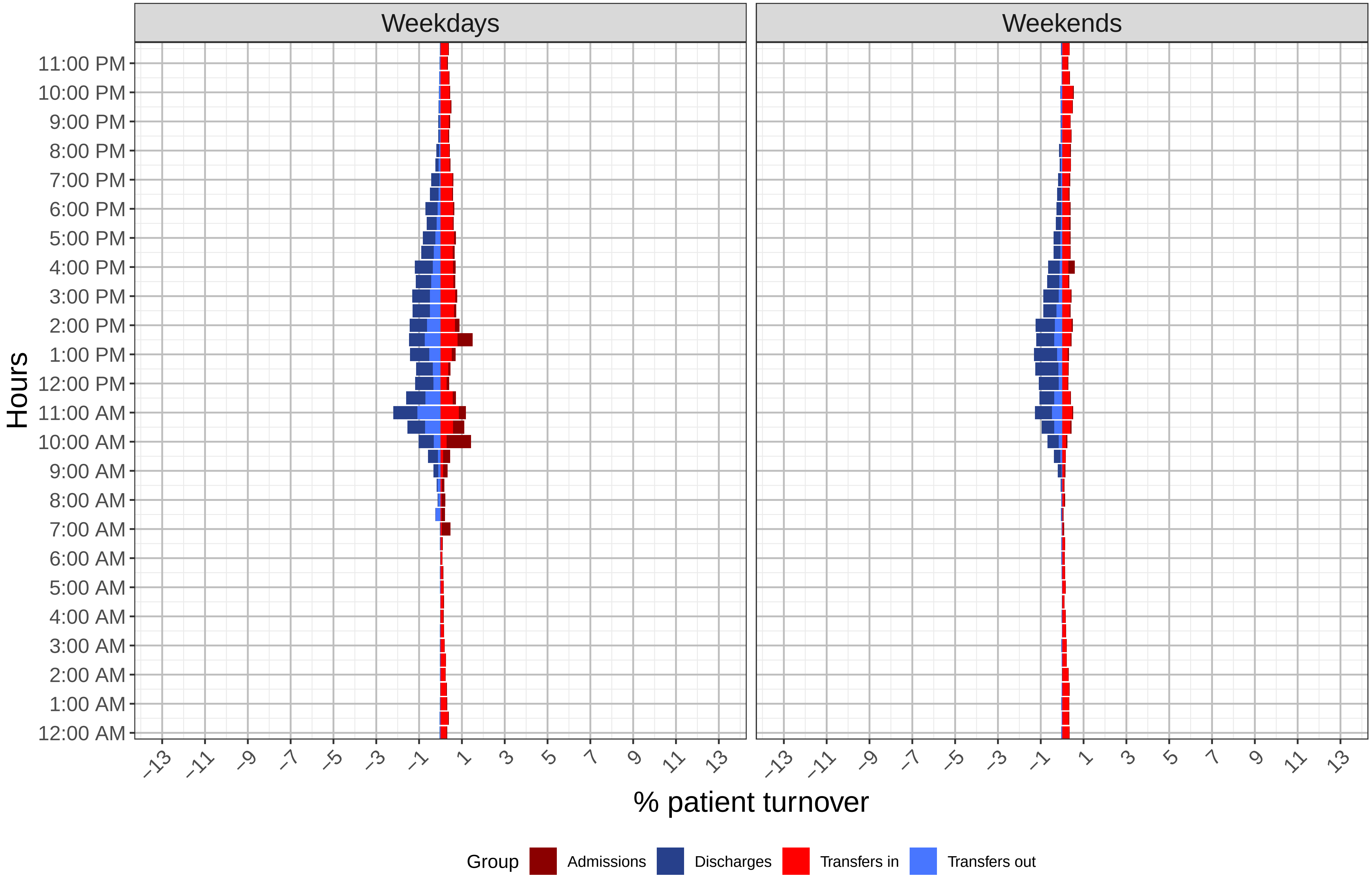

Intensive Care

Patient turnover percentages for the 48 data points split for weekdays and weekends

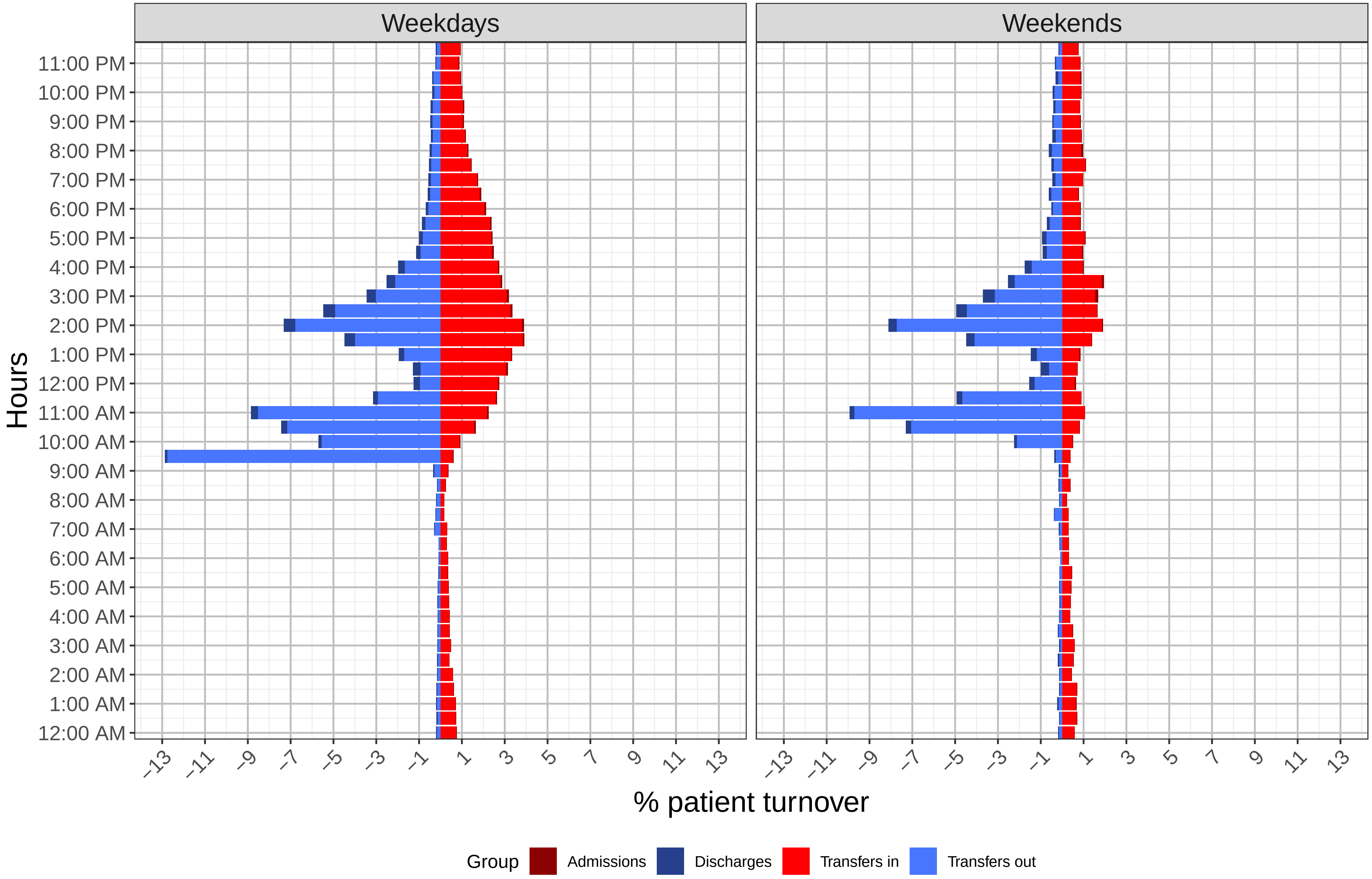

Supplement: Multimedia Appendix 5 [file jmir_v22i4e15554_app5.pdf]
